# Generation and Reactions of ε-Carbonyl Cations via Group 13 Catalysis

**DOI:** 10.3390/molecules27103078

**Published:** 2022-05-11

**Authors:** Page M. Penner, James R. Green

**Affiliations:** Department of Chemistry and Biochemistry, University of Windsor, Windsor, ON N9B 3P4, Canada; pagepenner@gmail.com

**Keywords:** ε-carbonyl cations, catalysis, umpolung, electrophilic aromatic substitution, allylation

## Abstract

The generation of ε-carbonyl cations and their reactions with nucleophiles is accomplished readily without transition metal cation stabilization, using the ε-bromide dienoate or dienone starting materials and GaCl_3_ or InCl_3_ catalysis. Arene nucleophiles are somewhat more straightforward than allyltrimethylsilane, but allyltrimethylsilane and propiophenone trimethysilyl enol ether each react successfully with InCl_3_ catalysis. The viability of these cations is supported by DFT calculations.

## 1. Introduction

**Figure 1:**
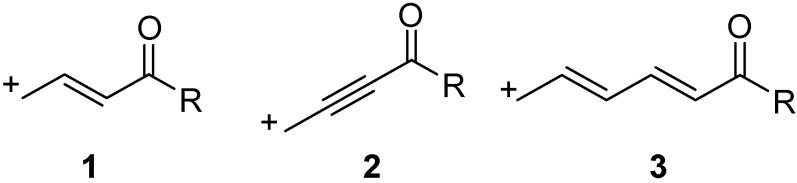
γ- and ε-carbonyl cations.

The reaction of electrophilic allyl and propargyl compounds with nucleophiles is a commonly used technique in organic chemistry. However, this chemistry becomes challenging when the system involves an electron-withdrawing group, such as a carbonyl. As a result, the generation and reaction of cations at the site γ- to a carbonyl or carbonyl equivalent (**1** and **2**) has seen only limited work, although it constitutes a fundamental form of umpolung chemistry (Figure 1). A modest but growing number of methods have been developed to obtain synthetic equivalents of these species. Propargyldicobalt [1] and allyliron [2] cations bearing electron withdrawing groups have been successfully generated and reacted with nucleophiles at the γ-site and are highly electrophilic. Activated cyclopropanes may serve as γ-carbonyl cation equivalents in the presence of Lewis acids, and they have close to the same level of electrophilicity [3]. Allylpalladiums and -iridiums bearing EWG’s are significantly less electrophilic but act catalytically and react well with stronger nucleophiles [4,5,6,7,8]. Methods giving an equivalent overall transformation, involving cationic species but not γ-carbonyl cations themselves, are known [9]. Nevertheless, methodology involving direct generation of γ-carbonyl cations without additional stabilization has remained elusive.

**Figure 2:**
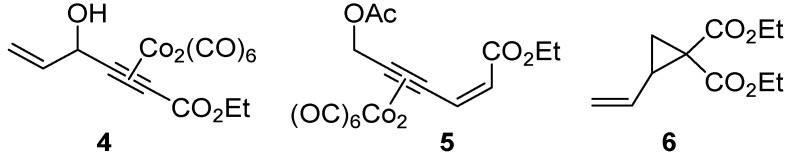
Existing ε-carbonyl cation equivalent precursors.

Research on vinylogous versions of γ-carbonyl cations, specifically on equivalents of ε-carbonyl cation equivalents (**3**), is still more scattered (Figure 2). The Green group has reported vinylogous Nicholas reactions involving compounds **4**–**5** to functionalize the site ε- to the carbonyl or carbonyl equivalent [10]. Activated vinylcyclopropanes (**6**) can, in principle, serve as ε-carbonyl cation equivalents, but Lewis acid mediated openings of these systems often favor reaction at the γ-site [3,11,12,13]. Transition metal mediated couplings are, in general, ε-selective, but again are only modestly electrophilic [14,15,16,17,18,19,20,21]. As a consequence, there the is an absence of work on ε-carbonyl cations or their equivalents that features both catalysis and high electrophilicity. Furthermore, the existence of a number of natural products containing ε-arylated carbonyls indicates significant synthetic utility to any methods capable of accessing such cations [22,23,24]. Unlike the γ-carbonyl cations themselves, the further conjugation possible to ε-carbonyl potentially ameliorates the effect of the electron-withdrawing group. As a result, we considered it worth investigating whether the ε-carbonyl cations themselves (**3**) could be generated, and whether this would be amenable to Lewis acid catalysis.

## 2. Results

**Scheme 1:**
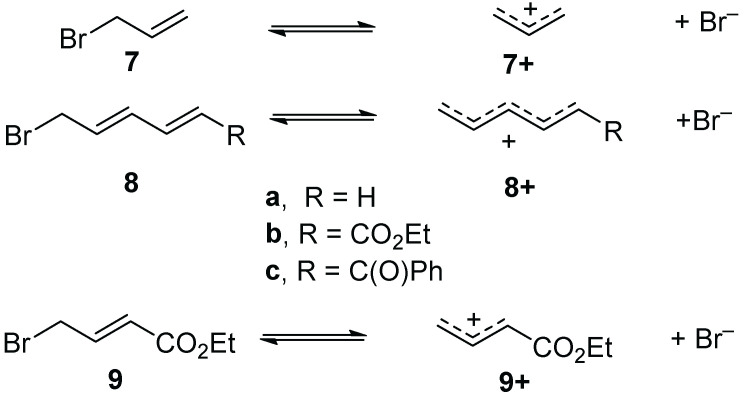
Allyl- and dienyl bromide ionization reactions.

The viability of direct generation of ε-carbonyl cations was initially addressed computationally, using DFT calculations employing the B3LYP functional and 6-311++G(d,p) basis set. The allyl bromide (**7**) to allyl cation (**7+**) transformation was the benchmark with which to compare results, as the viability of experimentally verified allyl cation synthetic chemistry has been established, most notably with indium(III) and related catalysts [25,26,27,28]. Compared to this was ionization of 5-bromo-1,3-pentadiene (**8a**) to give pentadienyl cation (**8a+**), and the analogous ionizations of ethyl 6-bromosorbate (**8b**), 6-bromo-1-phenyl-2,4-butan-1-one (**8c**). In addition, the ethyl 4-bromocrotonate (**9**) to γ-carbonyl cation species **9+** transformation was included, as an example of a process that has proven difficult experimentally (Scheme 1, Table 1).

The results of the calculations were promising. The ionization energy of **8a** to dienyl cation **8a+** was unsurprisingly the most favored, the process being 16.8 kcal/mol lower in energy than allyl cation generation. Somewhat to our surprise, the ionizations of the ε-carbonyl cation precursors **8b** and **8c** also were found to be favored substantially (by 10.9 kcal and 12.7 kcal, respectively), relative to the process with allyl bromide. Finally, the analogous ionization of ethyl 4-bromocrotonate was found to be 6.9 kcal/mol higher in energy than that of allyl bromide, consistent with the difficulty in discrete generation of γ-carbonyl cations. As a result of these findings, we chose to test these observations with an experiment. Given the notably mild conditions reported in the group 13 catalyzed electrophilic reactions of allyl bromides [25,26,27,28], we chose to pursue the analogous approach for ε-carbonyl cations.

**Scheme 2:**
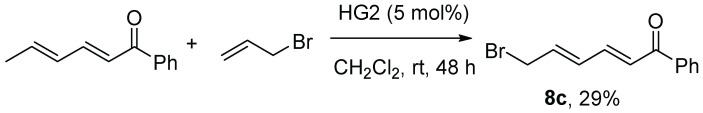
Preparation of phenyl ketone **8c**.

The ester- and phenyl ketone-substituted dienyl bromides, **8b**–**8c**, were chosen as substrates. Ethyl 6-bromohexadienoate (ethyl 6-bromosorbate, **8b**) was obtained by literature radical bromination of ethyl sorbate [29]. Phenyl ketone **8c** was prepared from 1-phenyl-2,4-butadienone [30], by HG-II-induced cross metathesis with allyl bromide (Scheme 2) [31].

**Scheme 3:**
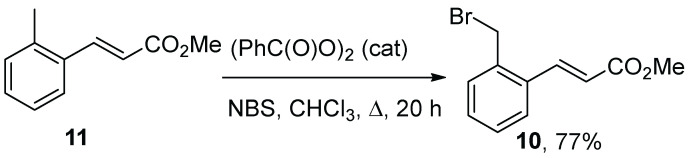
Preparation of benzylic bromide **10**.

In addition, a third substrate chosen for the study was **10**, employing an aryl spacer rather than one of the alkene spacers between the ester and bromide. Compound **10** was prepared by the radical bromination of cinnamate ester derivative **11** (**10**, 77%) (Scheme 3), itself being prepared by the Wittig reaction of *o*-tolualdehyde [32].

**Scheme 4:**
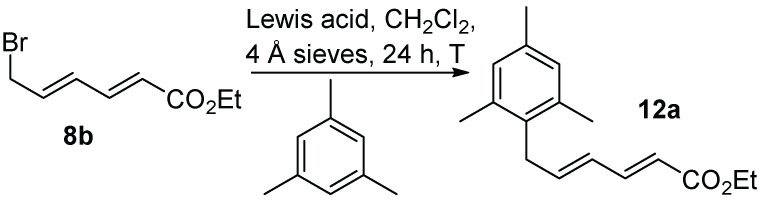
Formation of **12a** as a model reaction.

Experimental work began with ethyl 6-bromohexadienoate (ethyl 6-bromosorbate, **8b**). Test reactions were undertaken with mesitylene (5 equiv) as the nucleophile, and catalytic amounts (10 mol%) of Lewis acids CuCl, SnCl_4_, InCl_3_, GaCl_3_, and BiI_3_, in CH_2_Cl_2_ with 4 Å molecular sieves (Table 2, Scheme 4). CuCl and BiI_3_ afforded no product and minimal amounts of product, respectively. Conversely, GaCl_3_, InCl_3_, and SnCl_4_ gave more significant amounts of conversion to **12a** over 24 h, although small amounts of starting material remained. Repetition of the reactions at reflux afforded complete starting material consumption, but also gave some polar decomposition byproduct. Ultimately, GaCl_3_ at room temperature proved to be the most successful Lewis acid, giving **12a** in a 68% yield. Reducing the amount of GaCl_3_ to 5 mol% decreased the yield noticeably (47%), while an increase to 15 mol% made a negligible difference (67% yield). Omission of the 4 Å molecular sieves also gave a decrease in the yield of **12a** (51%, 58% brsm).

The characterization of **12a** was most clearly defined from the ^1^H NMR spectrum, which revealed a doublet (*J* = 15.4 Hz) at 5.77 ppm (H_α_), a doublet of doublets (*J* = 15.4, 11.0 Hz) at 7.30 ppm (H_β_), a doublet of doublets (*J* = 15.2, 11.0 Hz) at 6.02 ppm (H_γ_), and doublet of triplets (*J* = 15.2, 5.7 Hz) at 6.23 ppm (H_δ_), indicative of the conjugated diene of (*E*, *E*-) geometry resulting from ε-substitution. A small amount (<5% of the mixture) of isomeric material was co-eluted with the main product. Most of the ^1^H NMR spectral resonances are obscured by the dominant isomer due to the similar ^1^H spectral features, but with the H_ε_ methylene observable as a doublet of doublets (*J* = 7.4, 1.5 Hz) at 3.65 ppm, and with the H_β_ observable as a doublet of doublets (*J* = 15.1, 11.6 Hz) at 7.85 ppm, we have assigned this minor compound as the (2*E*, 4Z)-isomer of **12a**.

**Scheme 5:**
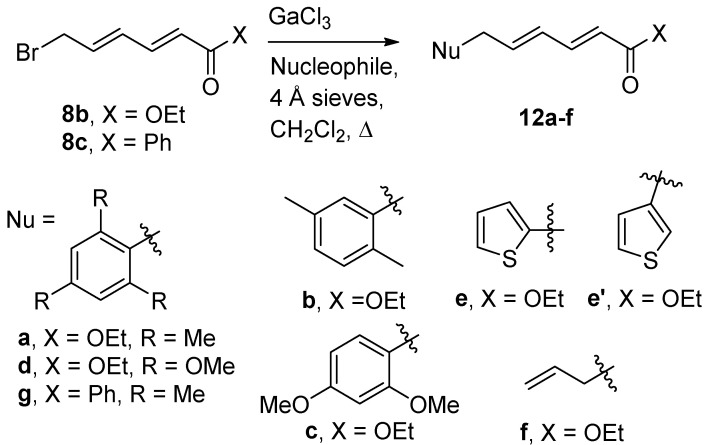
Reactions of ε-bromo dienyl carbonyls **8b**, **8c**.

These conditions were adopted for other arene nucleophiles, with the exception that the yields were found to be, in general, superior for other nucleophiles at reflux (Scheme 5, Table 3). *p*-Xylene, under analogous conditions, gave a modest yield of **12b** at rt (33% yield, 54% brsm), but better yields (65%) at reflux. 1,3-Dimethoxybenzene gave **12c** in 56% yield at reflux, while 1,3,5-trimethoxybenzene required 20 mol% GaCl_3_ for complete conversion, giving **12d** in 51% yield. Thiophene gave a 63% yield of product, as a 72:28 mixture C-2 (**12e**) and C-3 (**12e′**) substitutions. With allyltrimethylsilane, no condensation product was observed with 10 mol% GaCl_3_. Switching the catalyst to InCl_3_ was much more successful; 10 mol% InCl_3_ in CH_2_Cl_2_ at reflux gave approximately 80% conversion and 53% of **12f**, while 20 mol% InCl_3_ gave **12f** in a 66% yield. Finally, the phenyl ketone **8c** and mesitylene with GaCl_3_ at reflux gave **12g** in a 50% yield.

**Scheme 6:**
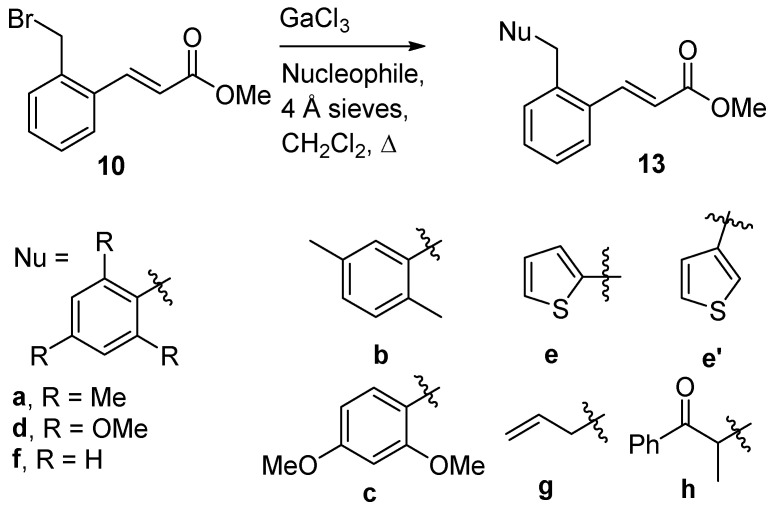
Reactions of ε-bromo aryl alkenoate **10**.

The benzylic bromide analogue, **10**, also reacted under the optimized conditions, again at reflux (Scheme 6, Table 4). Mesitylene afforded **13a** in a 73% yield, with no evidence of even trace amounts of isomeric products present. *p*-Xylene (**13b**, 76% yield), 1,3-dimethoxybenzene (**13c**, 77% yield), and 1,3,5-trimethoxybenzene (**13d**, 75% yield) behaved analogously. Thiophene worked well, again affording an isomeric mixture of C-2 and C-3 substitution products (**13e** and **13e′**, 92% yield, **13e**:**13e′** = 71:29). The aromatic nucleophiles could be extended to benzene itself (**13f**, 72% yield), although a greater amount of GaCl_3_ catalyst (30 mol%) was required.

The reaction with allyltrimethylsilane was again more difficult than for arene nucleophiles with GaCl_3_ catalysis. In this case, while 10 mol% GaCl_3_ showed no significant conversion, 50 mol% GaCl_3_ gave a 46% yield of **13g**. InCl_3_ again proved to be a superior catalyst with allyltrimethylsilane; 10 mol% of InCl_3_ afforded a 29% yield of **13g**, while raising the catalyst amount to 20 mol% InCl_3_ gave **13g** in 64% (78% brsm). Finally, a switch to higher temperature reaction conditions (1,2-dichloroethane, reflux) demonstrated that propiophenone trimethylsilyl enol ether was also amenable to reaction with **10** (**13h**, 82% yield) with the use of InCl_3_ as the catalyst.

## 3. Discussion

An analysis of the results suggests several issues worth discussing. First of all, despite the unmanageable superficial appearance of ε-carbonyl cations, they are quite viable. Transition metal stabilization of the cationic dienyl (or enynyl) unit is not mandatory. The use of dienyl bromides and Ga(III) or In(III) catalysts is capable of generating ε-carbonyl cations that react with nucleophiles in moderate yields with **8b**–**c**, and in good yields with **10**. The reactions require somewhat more vigorous conditions than with allyl bromide itself, and we attribute this to the presence of the Lewis basic carbonyl functions in the substrates, and in some cases, the reacting nucleophiles. Arene nucleophiles react with greater facility than allylsilanes using GaCl_3_, although conditions can normally be found using InCl_3_ that give synthetically useful yields of **12f** and **13g**. InCl_3_ also allows the successful reaction of an enol silane (**13h**). The successful incorporation of benzene as a nucleophile (**13f**) indicates that the current protocol can allow incorporation of less reactive nucleophiles than the Nicholas reaction-based ε-carbonyl cation equivalents [10] and far less reactive nucleophiles than the analogous transition metal catalyzed equivalents [14,15,16,17,18,19,20,21]. The question of competitive conjugate addition does not appear problematic with the arene, allylsilane, or enol silane nucleophiles. For example, the crude reaction product of **8b** and allyltrimethylsilane showed no evidence of conjugate addition byproducts. Conversely, trial reactions with triethylsilane, a substantially stronger nucleophile than arenes or allyltrimethylsilane [33], appeared to give mixtures whose ^1^H NMR spectra included multiple aliphatic resonances, suggesting the conjugate addition may be a major reaction pathway there.

## 4. Materials and Methods

The starting materials and reagents involved in the reactions were purchased from commercial sources, unless otherwise noted. GaCl_3_ and InCl_3_ were stored under an inert atmosphere prior to use. Purification of synthesized products was conducted by either column chromatography (using SilaFlash^®^ P60, 230–400 mesh, SiliCycle, Quebec City, QC, Canada), preparative TLC (SiliaPlate, 1000 μm thickness, SiliCycle, Quebec City, QC, Canada) or radial chromatography (Silica gel, 2000 μm thickness, EM Science, Gibbstown, NJ, USA). Analytical thin layer chromatography (TLC) was performed using Silicycle aluminum-backed sheets (SiliCycle, Quebec City, QC, Canada). Dichloromethane and tetrahydrofuran solvents (Sigma-Aldrich Canada, Milton, ON, Canada) were obtained from a solvent purification system. All of the reactions were performed under an atmosphere of nitrogen unless otherwise stated. Prior to reaction, all glassware was dried in an oven at 110 °C for a minimum of one hour and subsequently cooled in a desiccator. Reactions conducted at greater than 25 °C were conducted in a heated oil bath.

All of the NMR spectral analyses were conducted on 300 MHz and 500 MHz spectrometers (Bruker Canada, Milton, ON, Canada) at room temperature in solutions of CDCl_3_ (CIL, Andover, MA, USA). The residual CHCl_3_ peak was set to 7.27 ppm and 77.0 ppm for the ^1^H NMR and ^13^C NMR spectra, respectively. ^1^H NMR spectral data are listed with units of ppm for peak position (δ) and Hz for coupling constant (*J*). The following symbols were used for peak appearance: s, singlet; d, doublet; t, triplet; dd, doublet of doublets; dt, doublet of triplets; q, quartet; m, multiplet. The ^1^H and ^13^C NMR spectra are available in the Supplementary Materials. The IR analysis was conducted on an ATR infrared (FTIR) spectrometer (Bruker Canada, Milton, ON, Canada). For IR spectra listed in the characterization of compounds and the absorption peaks with the greatest functional group relevance are reported in wavenumbers (cm^−1^). High resolution mass spectrometry results were obtained by direct insertion probe on a Waters Xevo G2-XS Time-of-Flight Mass Spectrometer (Waters, Toronto, ON, Canada) in ASAP(+) mode at the University of Windsor Mass Spectrometry lab. The computational calculations were conducted with Gaussview 5.0.9 and B3LYP/6-311++G(d,p) to optimize the structures studied, both with and without solvation in dichloromethane. Final coordinates are available in the Supplementary Materials.

### 4.1. 6-Bromo-1-phenyl-2,4-hexadienone (***8c***)

A procedure for synthesis of similar compounds had previously been reported, [31] so this procedure was adapted to use on 1-phenyl-2,4-hexadienone. To a solution of 1-phenyl-2,4-hexadienone (0.2287 g, 1.33 mmol) and allyl bromide (0.56 mL, 6.6 mmol, 5 equiv.) in dichloromethane (40 mL) were added to the Hoveyda-Grubbs II catalyst (0.021 g, 0.034 mmol, 2.5 mol%). After stirring under N_2_ for 24 h, another portion of Hoveyda Grubbs II catalyst (0.021 g, 0.034 mmol, 2.5 mol%) was added. After 48 h total, the solvent was evaporated under reduced pressure and the product was subjected to flash chromatography (5:1 PE:Et_2_O) to yield **8c** as a yellow solid (0.0982 g, 29%). **IR** (neat) λ_max_ 3024, 2921, 2856, 1660, 1261, 1003, 693, and 590 cm^−1^; **^1^H NMR** (00 MHz, CDCl_3_) δ 7.95 (d, *J* = 8.7 Hz, 2H), 7.58 (apparent t, *J* = 7.4 Hz, 1H), 7.49 (apparent t, *J* = 7.6 Hz, 2H), 7.39 (dd, *J* = 15.1, 11.0 Hz, 1H), 7.02 (d, *J* = 15.1 Hz, 1H), 6.53 (dd, *J* = 15.0 Hz, 11.0 Hz, 1H), 6.36 (m, 1H), and 4.07 (d, *J* = 7.7 Hz, 2H); **^13^C NMR** (125 MHz, CDCl_3_) δ 190.2, 142.5, 137.8, 132.9, 132.6, 128.6, 128.4, 127.0, and 31.3; the **HRMS** m/e for C_12_H_11_BrO calculated (M + 1)^+^ 251.0072, found 251.0068.

### 4.2. Methyl 3-[2-(Bromomethyl)phenyl]acrylate (***10***)

Bromination was conducted with methods derived from those described by Snead [34]. Methyl 3-(2-methylphenyl)acrylate **11** (1.1761 g, 4.2 mmol) and N-bromosuccinimide (1.6947 g, 9.522 mmol) were heated to reflux in chloroform (35 mL). Once at reflux, benzoyl peroxide (0.1670 g, 0.6894 mmol) was added. The reaction was stirred at reflux for 20 h, then cooled, filtered through Celite^®^ (Sigma-Aldrich Canada, Milton, ON, Canada) and concentrated under reduced pressure. The residue was then subjected to flash chromatography (10:1 petroleum ether: Et_2_O) and 0.8078 g (77%) of light yellow solid product **10** was obtained. The mp was 84.5–85.5 °C. **IR** (neat) λ_max_ 3030, 2950, 1700, 1431, 1078, and 599 cm^−1^; **^1^H NMR** (300 MHz, CDCl_3_) δ 8.03 (d, *J* = 15.9 Hz, 1H), 7.53 (m, 1H), 7.30 (m, 3H), 6.40 (d, *J* = 15.9 Hz, 1H), 4.54 (s, 2H), and 3.78 (s, 3H); **^13^C NMR** (75 MHz, CDCl_3_) δ 166.7, 140.5, 136.4, 133.4, 130.5, 130.1, 129.1, 127.0, 120.4, 51.6, and 30.4; the **HRMS** m/e for C_11_H_11_BrO_2_ calculated (M + 1)^+^ 255.0021, and found 255.0019.

### 4.3. Ethyl 6-(2,4,6-Trimethylphenyl)-2,4-hexadienoate (***12a***)

To a suspension of GaCl_3_ (0.009 g, 0.05 mmol, 10 mol%) and 4Å molecular sieves (ca. 0.4 g), CH_2_Cl_2_ (6 mL) was added to mesitylene (0.37 mL, 2.67 mmol, 5 equiv.) and **8b** (0.1161 g, 0.5299 mmol) at room temperature. The reaction was stirred under N_2_ and monitored by TLC for 26 h. Following removal of volatiles under reduced pressure and flash chromatography (10:1 PE:Et_2_O), **12a** (0.0902 g, 68%) was isolated as a yellow oil. This compound was also made by methods outlined below in General Procedure 1, where the reaction was brought to reflux for 22 h after the reagents were added. This afforded the product **12a** in a 63% yield. **IR** (neat) λ_max_ 2975, 2919, 2861, 1709, 1638, and 1130 cm^−1^; **^1^H NMR** (500 MHz, CDCl_3_) δ 7.30 (dd, *J* = 15.4 Hz, 11.0 Hz, 1H), 6.90 (s, 2H), 6.23 (dt, *J* = 15.2 Hz, 5.7 Hz, 1H), 6.02 (dd, *J* = 15.2 Hz, 11.0 Hz, 1H), 5.77 (d, *J* = 15.4 Hz, 1H), 4.21 (q, *J* = 7.2 Hz, 2H), 3.51 (d, *J* = 5.7 Hz, 2H), 2.31 (s, 3H), 2.28 (s, 6H), and 1.31 (t, *J* = 7.1 Hz, 3H); **^13^C NMR** (125 MHz, CDCl_3_) δ 167.0, 144.5, 141.0, 136.5, 135.8, 131.9, 128.9, 128.1, 119.6, 60.0, 32.5, 20.8, 19.7, and 14.2; the **HRMS** m/e for C_17_H_22_O_2_ calculated (M + 1)^+^ 259.1698, and found 259.1691.

### 4.4. Ethyl 6-(2,5-Dimethylphenyl)-2,4-hexadienoate (***12b***)

**General Procedure 1.** To a suspension of GaCl_3_ (0.004 g, 0.02 mmol, 10 mol%) and 4Å molecular sieves (ca. 0.4 g), CH_2_Cl_2_ (6 mL) was added to para-xylene (0.14 mL, 1.1 mmol, 5 equiv.) and **8b** (0.048 g, 0.22 mmol) at room temperature. The mixture was heated to reflux, stirred under N_2_ and monitored by TLC for 23 h. Following removal of volatiles under reduced pressure and flash chromatography (5:1 PE:Et_2_O), **12b** (0.0349 g, 65%) was isolated as a yellow oil. This compound was also prepared where the reaction was stirred at room temperature for 23 h, and the yield of product **12b** was 34%. **IR** (neat) λ_max_ 2979, 2925, 1710, 1640, 1131, 1000, and 810 cm^−1^; **^1^H NMR** (300 MHz, CDCl_3_) δ 7.29 (dd, *J* = 15.3 Hz, 10.5 Hz, 1H), 7.06 (d, *J* = 7.5 Hz, 1H), 6.96 (m, 2H), 6.26 (dt, *J* = 15.3 Hz, 6.0 Hz, 1H), 6.12 (dd, *J* = 15.9 Hz, 10.5 Hz, 1H), 5.80 (d, *J* = 15.0 Hz, 1H), 4.20 (q, *J* = 7.2 Hz, 2H), 3.46 (d, *J* = 6.3 Hz, 2H), 2.31 (s, 3H), 2.24 (s, 3H), and 1.29 (t, *J* = 6.9 Hz, 3H); **^13^C NMR** (75 MHz, CDCl_3_) δ 167.0, 144.4, 141.6, 136.5, 135.4, 132.9, 130.0, 129.8, 128.9, 127.1, 119.7, 60.0, 36.6, 20.7, 18.7, and 14.1; the **HRMS** m/e for C_16_H_20_O_2_ calculated (M + 1)^+^ 245.1550, and found 245.1539.

### 4.5. Ethyl 6-(2,4-Dimethoxyphenyl)-2,4-hexadienoate (***12c***) 

General Procedure 1 was carried out with GaCl_3_ (0.005 g, 0.030 mmol, 10 mol%), 1,3-dimethoxybenzene (0.20 mL, 1.5 mmol, 5 equiv.) and **8b** (0.0653 g, 0.298 mmol). The reaction was monitored by TLC for 23 h under reflux and N_2_, and after purification by flash chromatography (3:1 PE:Et_2_O), **12c** (0.0460 g, 56%) was isolated as a yellow oil. **IR** (neat) λ_max_ 2935, 2837, 1708, 1207, 1155, 1132, and 1035 cm^−1^; **^1^H NMR** (300 MHz, CDCl_3_) δ 7.28 (dd, *J* = 10.8 Hz, 5.1 Hz, 1H), 7.00 (d, *J* = 7.5 Hz, 1H), 6.50 (m, 2H), 6.21 (m, 2H), 5.78 (d, *J* = 15.3 Hz, 1H), 4.19 (q, *J* = 7.2 Hz, 2H), 3.80 (s, 6H), 3.42 (d, *J* = 6.6 Hz, 2H), and 1.29 (t, *J* = 6.9 Hz, 3H); **^13^C NMR** (75 MHz, CDCl_3_) δ 167.1, 159.5, 157.9, 144.8, 142.6, 129.9, 128.4, 119.3, 103.8, 98.4, 59.9, 55.2, 32.8, and 14.1; the **HRMS** m/e for C_16_H_20_O_4_ calculated (M + 1)^+^ 277.1440, and found 277.1440.

### 4.6. Ethyl 6-(2,4,6-Trimethoxyphenyl)-2,4-hexadienoate (***12d***)

General Procedure 1 was carried out with GaCl_3_ (0.010 g, 0.057 mmol, 20 mol%), 1,3,5-trimethoxybenzene (0.2521 g, 1.499 mmol, 5 equiv.) and **8b** (0.0629 g, 0.287 mmol). The reaction was monitored by TLC for 24 h under reflux and N_2_, and after purification by flash chromatography (3:1 PE:Et_2_O), **12d** (0.0446 g, 51%) was isolated as a beige solid, and the mp was 69–70.5 °C. **IR** (neat) λ_max_ 2941, 2837, 1697, 1595, and 1149 cm^−1^; **^1^H NMR** (500 MHz, CDCl_3_) δ 7.25 (dd, *J* = 15.3 Hz, 11.0 Hz, 1H), 6.22 (dt, *J* = 15.1, 6.4 Hz, 1H), 6.15 (s, 2H), 6.10 (m, 2H), 5.74 (d, *J* = 15.3 Hz, 1H), 4.17 (q, *J* = 7.2 Hz, 2H), 3.82 (s, 3H), 3.80 (s, 6H), 3.43 (d, *J* = 6.4 Hz, 2H), and 1.27 (t, *J* = 7.2 Hz, 3H); **^13^C NMR** (75 MHz, CDCl_3_) δ 167.3, 159.8, 158.7, 145.5, 143.2, 127.6, 118.8, 107.6, 90.6, 60.0, 55.7, 55.3, 26.1, and 14.3; the **HRMS** m/e for C_17_H_22_O_5_ calculated (M + 1)^+^ 307.1545, and found 307.1539.

### 4.7. Ethyl 6-(2-Thienyl)-2,4-hexadienoate (***12e***) and Ethyl 6-(3-thienyl)-2,4-hexadienoate (***12e′***)

General Procedure 1 was carried out with GaCl_3_ (0.004 g, 0.02 mmol, 10 mol%), thiophene (0.17 mL, 2.1 mmol, 10 equiv.) and **8b** (0.0476 g, 0.217 mmol). The reaction was monitored by TLC for 23 h under reflux and N_2_, and after purification by flash chromatography (4:1 PE:Et_2_O), an **12e**/**12e′** mixture (0.0306 g, 63%) was isolated as a yellow oil. The product contained a 72:28 **12e**:**12e′** based on ^1^H NMR spectral integration of the resonances at 3.70 ppm (**12e**), and 3.52 ppm (**12e′**) corresponding to the hydrogen atoms bonded to the sp^3^ carbon adjacent to the thiophene, but these two compounds were not able to be separated. **IR** (neat) λ_max_ 2980, 2934, 1707, 1253, and 1131 cm^−1^; **^1^H NMR** (500 MHz, CDCl_3_) δ 7.25–7.34 (m, 1H), 7.18 (d, *J* = 5.1 Hz, 1H), 6.96 (dd, 5.1, 3.5 Hz, 1H), 6.83 (m, 1H), 6.19–6.31 (m, 2H), 5.86 (d, *J* = 15.0 Hz, 1H), 4.21 (q, *J* = 7.2 Hz, 2H), 3.70 (d, *J* = 5.7 Hz, 2H), and 1.30 (t, *J* = 7.2 Hz, 3H). Resonances from minor product **12e′** were observed at: δ 6.98 (m, 1H), 6.93 (dd, *J* = 4.9, 1.2 Hz, 1H), 5.83 (d, *J* = 15.3 Hz, 1H), and 3.52 (d, *J* = 5.4 Hz, 2H); **^13^C NMR** (125 MHz, CDCl_3_) δ 167.0, 144.0, 141.3, 140.6, 129.5, 127.0, 125.8, 125.1, 124.0, 120.8, 60.3, 33.1, and 14.3. Resonances from minor product **12e′** were observed at: δ 167.1, 144.3, 139.0, 129.3, 128.1, 125.8, 121.2, 120.3, 60.2, and 33.7; the **HRMS** m/e for C_12_H_14_O_2_S calculated (M + 1)^+^ 223.0793, and found 223.0797.

### 4.8. Ethyl 2,4,6-Nonatrienoate (***12f***)

A mixture of InCl_3_ (0.0127 g, 20 mol%), 4Å molecular sieves, **8b** (0.0633 g, 0.289 mmol) and allyltrimethylsilane (0.23 mL, 5 equiv) in CH_2_Cl_2_ (7 mL) were heated to reflux under N_2_ for 14 h. Following a conventional workup, preparative TLC (7.5:1 hexanes: Et_2_O) afforded **12f** (0.0343 g, 66%) as a faintly tan oil. **IR** (neat) λ_max_ 2980, 2928, 1712, 1253, 1136, and 998 cm^−1^; **^1^H NMR** (500 MHz, CDCl_3_) δ 7.25 (dd, *J* = 15.4 Hz, 10.5 Hz, 1H), 6.19 (d of ½ AB, *J* = 10.5, 15.2 Hz, 1H), 6.11 (t of ½ AB, *J* = 6.5, 15.2 Hz, 1H), 5.73–5.84 (m, 2H), 5.03 (dd, *J* = 17.1, 1.6 Hz, 1H), 4.99 (dd, *J* = 10.2, 1.6 Hz, 1H), 4.19 (q, *J* = 7.2 Hz, 2H), 2.27 (m, 2H), 2.19 (m, 2H), and 1.28 (t, *J* = 7.2 Hz, 3H). Resonances from the minor (2*E*, 4*Z*) isomer can be observed at 6.89 (dd, *J* = 15.7, 7.5 Hz, 1H), 5.73 (m, 1H), 5.10 (d, *J* = 10.3 Hz, 1H), and 4.18 (obscured q, *J* = 7.1 Hz, 2H); **^13^C NMR** (125 MHz, CDCl_3_) δ 167.2, 144.8, 143.3, 137.4, 128.7, 119.5, 115.3, 60.1, 32.7, 32.2, and 14.3; the **HRMS** m/e for C_11_H_16_O_2_ calculated (M + 1)^+^ 181.1228, and found 181.1228.

### 4.9. 6-(2,4,6-Trimethylphenyl)-1-phenyl-2,4-hexadienone (***12g***)

To a suspension of GaCl_3_ (0.003 g, 0.02 mmol, 10 mol%), and 4Å molecular sieves (ca. 0.4 g), CH_2_Cl_2_ (6 mL) was added to mesitylene (0.12 mL, 0.86 mmol, 5 equiv.) and **8c** (0.0438 g, 0.17 mmol) at room temperature. The reaction was heated to reflux, stirred under N_2_ and monitored by TLC for 20 h. Following the removal of volatiles under reduced pressure and flash chromatography (10:1 PE:Et_2_O), **12g** (0.0251 g, 50%) was isolated as a yellow oil. **IR** (neat) λ_max_ 3000, 2917, 2851, 1660, 1587, 1000, 693 cm^−1^; **^1^H NMR** (300 MHz, CDCl_3_) δ 7.92 (d, *J* = 7.2 Hz, 2H), 7.55 (m, 1H), 7.36–7.50 (m, 3H), 6.90 (s, 2H), 6.83 (d, *J* = 15.0 Hz, 1H), 6.37 (dt, *J* = 15.0 Hz, 5.7 Hz, 1H), 6.10 (dd, *J* = 15.0 Hz, 11.1 Hz, 1H), 3.54 (d, *J* = 5.1 Hz, 2H), 2.30 (s, 3H), and 2.27 (s, 6H); **^13^C NMR** (75 MHz, CDCl_3_) δ 190.8, 144.9, 143.0, 138.2, 136.5, 135.9, 132.5, 131.9, 129.0, 128.9, 128.5, 128.3, 124.0, 32.8, 20.9, and 19.8; the **HRMS** m/e for C_21_H_22_O calculated (M + 1)^+^ 291.1749, and found 291.1745.

### 4.10. Methyl 3-[2-(2,4,6-Trimethylbenzyl)phenyl]acrylate (***13a***)

**General procedure 2.** To a suspension of GaCl_3_ (0.004 g, 0.02 mmol, 10 mol%), and 4Å molecular sieves (ca. 0.4 g), CH_2_Cl_2_ (6 mL) was added to mesitylene (0.15 mL, 5 equiv.) and **10** (0.0532 g, 0.210 mmol) at room temperature. The reaction was heated to reflux, stirred under N_2_ and monitored by TLC for 24 h. Following removal of volatiles under reduced pressure and chromatography (5:1 PE:Et_2_O), **13a** (0.0449 g, 73%) was obtained as a beige solid; mp was 81.5–83.0 °C. **IR** (neat) λ_max_ 3056, 2969, 2948, 2915, 1713, 1164, 982, and 760 cm^−1^; **^1^H NMR** (500 MHz, CDCl_3_) δ 8.24 (d, *J* = 15.6 Hz, 1H), 7.59 (dd, *J* = 6.9 Hz, 2.1 Hz, 1H), 7.14–7.23 (m, 2H), 6.93 (s, 2H), 6.60 (d, *J* = 7.8 Hz, 1H), 6.44 (d, *J* = 15.9 Hz, 1H), 4.10 (s, 2H), 3.86 (s, 3H), 2.32 (s, 3H), and 2.15 (s, 6H); **^13^C NMR** (125 MHz, CDCl_3_) δ 167.4, 142.2, 139.1, 137.2, 135.9, 133.4, 132.7, 130.3, 128.9, 127.2, 126.5, 126.3, 119.5, 51.7, 31.8, 20.9, and 19.9; the **HRMS** m/e for C_20_H_23_O_2_ calculated (M + 1)^+^ 295.1698, and found 295.1699.

### 4.11. Methyl 3-[2-(2,5-Dimethylbenzyl)phenyl]acrylate (***13b***)

General procedure 2 was carried out with GaCl_3_ (0.004 g, 0.02 mmol, 10 mol%), para-xylene (0.13 mL, 5 equiv.) and **10** (0.0540 g, 0.213 mmol). The reaction was monitored by TLC for 21 h under reflux and N_2_, and after evaporation under reduced pressure and purification by flash chromatography (5:1 PE:Et_2_O), **13b** (0.0452 g, 76%) was obtained as a faintly yellow solid; mp was 51.0–53.0 °C. **IR** (neat) λ_max_ 3015, 2949, 2923, 2892, 1714, 1172, 1015, 977, and 765 cm^−1^; **^1^H NMR** (500 MHz, CDCl_3_) δ 8.03 (d, *J* = 15.9 Hz, 1H), 7.61 (dd, *J* = 7.5 Hz, 1.5 Hz, 1H), 7.24–7.32 (m, 2H), 7.10, (d, *J* = 7.8 Hz, 1H), 6.98 (m, 2H), 6.75 (s, 1H), 6.38 (d, *J* = 15.9 Hz, 1H), 4.07 (s, 2H), 3.81 (s, 3H), 2.26 (s, 3H), and 2.23 (s, 3H); **^13^C NMR** (125 MHz, CDCl_3_) δ 167.3, 142.4, 139.8, 137.7, 135.5, 133.5, 133.3, 130.4, 130.13, 130.11, 129.9, 127.2, 126.7, 126.6, 119.5, 51.7, 36.2, 21.0, and 19.1; the **HRMS** m/e for C_19_H_21_O_2_ calculated (M + 1)^+^ 281.1541, and found 281.1544.

### 4.12. Methyl 3-[2-(2,4-Dimethoxybenzyl)phenyl]acrylate (***13c***)

General procedure 2 was carried out with GaCl_3_ (0.003 g, 0.02 mmol, 10 mol%), 1,3-dimethoxybenzene (0.11 mL, 0.84 mmol, 5 equiv.) and **10** (0.0445 g, 0.175 mmol). The reaction was monitored by TLC for 22 h under reflux and N_2_, and after evaporation under reduced pressure and purification by flash chromatography (4:1 PE:Et_2_O), **13c** (0.0423 g, 77%) was isolated as a light yellow viscous oil. **IR** (neat) λ_max_ 2934, 2878, 2837, 1716, 1241, 1114, and 1036 cm^−1^; **^1^H NMR** (300 MHz, CDCl_3_) δ 8.11 (d, *J* = 15.9 Hz, 1H), 7.58 (dd, *J* = 7.5 Hz, 1.2 Hz, 1H), 7.20–7.34 (m, 2H), 7.17 (dd, *J* = 6.0 Hz, 1.2 Hz, 1H), 6.81 (d, *J* = 8.3 Hz, 1H), 6.48 (d, *J* = 2.4 Hz, 1H), 6.38 (dd, *J* = 8.3, 2.4 Hz, 2H), 6.36 (d, *J* = 15.9 Hz, 1H), 4.03 (s, 2H), 3.83 (s, 3H), 3.80 (s, 3H), and 3.79 (s, 3H); **^13^C NMR** (75 MHz, CDCl_3_) δ 167.4, 159.5, 157.9, 142.9, 140.7, 133.5, 130.6, 130.3, 130.0, 126.5, 126.4, 121.0, 119.0, 104.0, 98.4, 55.3, 51.6, and 32.1; the **HRMS** m/e for C_19_H_20_O_4_ calculated (M + 1)^+^ 313.1440, and found 313.1441. 

### 4.13. Methyl 3-[2-(2,4,6-Trimethoxybenzyl)phenyl]acrylate (***13d***)

General procedure 2 was carried out with GaCl_3_ (0.004 g, 0.02 mmol, 10 mol%), 1,3,5-trimethoxybenzene (0.1907 g, 1.134 mmol, 5 equiv.) and **10** (0.0547 g, 0.215 mmol). The reaction was monitored by TLC for 22 h under reflux and N_2_, and after evaporation under reduced pressure and purification by flash chromatography (5:1 PE:Et_2_O), **13d** (0.0552 g, 75%) was obtained as a colorless solid; the mp was 84–85 °C. **IR** (neat) λ_max_ 2949, 2839, 1702, 1118, 949, and 764 cm^−1^; **^1^H NMR** (300 MHz, CDCl_3_) δ 8.43 (d, *J* = 15.9 Hz, 1H), 7.53 (dd, *J* = 7.5 Hz, 1.2 Hz, 1H), 7.14–7.25 (m, 2H), 7.11 (m, 1H), 6.37 (d, *J* = 15.9 Hz, 1H), 6.17 (s, 2H), 4.06 (s, 2H), 3.84 (s, 3H), 3.83 (s, 3H), and 3.77 (s, 6H); **^13^C NMR** (75 MHz, CDCl_3_) δ 167.7, 159.9, 158.9, 143.8, 141.6, 133.1, 129.7, 129.3, 126.1, 125.8, 118.4, 109.0. 90.5, 55.5, 55.3, 51.5, and 25.5; the **HRMS** m/e for C_20_H_23_O_5_ calculated (M + 1)^+^ 343.1545, and found 343.1547.

### 4.14. Methyl 3-[2-(2-Methylthienyl)phenyl]acrylate (***13e***) and Methyl 3-[2-(3-methylthienyl)phenyl]acrylate (***13e′***)

General procedure 2 was carried out with GaCl_3_ (0.003 g, 0.02 mmol, 10 mol%), thiophene (0.075 mL, 0.94 mmol, 5 equiv.) and **10** (0.0465 g, 0.183 mmol). The reaction was monitored by TLC for 20 h under reflux and N_2_, and after purification by flash chromatography (5:1 PE:Et_2_O), the **13e**/**13e′** mixture (0.0437 g, 92% combined) was found as a light yellow oil. Based on ^1^H NMR integration of the hydrogen atoms bonded to the sp^3^ carbon adjacent to the thiophene group (4.27 ppm for **13e** and 4.10 ppm for **13e′**), the product is an inseparable mixture of **13e**:**13e′** in a ratio of 71:29. **IR** (neat) λ_max_ 2949, 1711, 1170, 977, 763, 731, and 698 cm^−1^; **^1^H NMR** (300 MHz, CDCl_3_) δ 8.02 (d, *J* = 15.6 Hz, 1H), 7.57 (d, *J* = 7.5 Hz, 1H), 7.28 (m, 3H), 7.12 (d, *J* = 5.1 Hz, 1H), 6.88 (m, 1H), 6.72 (d, *J* = 3.0 Hz, 1H), 6.34 (d, *J* = 15.9 Hz, 1H), 4.27 (s, 2H), and 3.78 (s, 3H). Most resonances from minor product **13e′** were superimposed on those from **13e,** but the following resonances from **13e′** were clearly observed: δ 6.83 (s, 1H), and 4.10 (s, 2H); **^13^C NMR** (75 MHz, CDCl_3_) δ 167.1, 143.0, 141.9, 139.5, 133.1, 130.3, 130.2, 127.2, 126.8, 126.7, 125.1, 124.0, 119.6, 51.6, and 33.3. Some resonances from minor product **13e′** were superimposed on those from **13e** but the following resonances from **13e′** were clearly observed: δ 142.2, 139.8, 133.2, 130.4, 130.1, 128.0, 126.9, 126.6, 125.7, 125.2, 121.4, 119.3, and 33.8; the **HRMS** m/e for C_15_H_14_O_2_S calculated (M + 1)^+^ 259.0793, and found 259.0801.

### 4.15. Methyl 3-[2-Benzylphenyl]acrylate (***13f***)

General procedure 2 was carried out with GaCl_3_ (0.0107 g, 0.061 mmol, 30 mol%), benzene (0.25 mL, 14 equiv.) and **10** (0.0518 g, 0.204 mmol). The reaction was monitored by TLC for 30 h under reflux and N_2_, and following a conventional (CH_2_Cl_2_) extractive workup and purification by preparative TLC (7:1 PE:Et_2_O); **13f** (0.0367 g, 72%) was obtained as a faintly tan oil. **IR** (neat) λ_max_ 3062, 3026, 2950, 1714, 1172, 1634, and 1599 cm^−1^; **^1^H NMR** (300 MHz, CDCl_3_) δ 8.06, (d, *J* = 15.8 Hz, 1H), 7.61 (d, *J* = 7.5 Hz, 1H), 7.10–7.40 (m, 8H), 6.36 (d, *J* = 15.8 Hz, 1H), 4.16 (s, 2H), and 3.80 (s, 3H); **^13^C NMR** (75 MHz, CDCl_3_) δ 167.2, 142.4, 140.14, 140.05, 133.5, 130.8, 130.1, 128.7, 128.5, 126.9, 126.7, 126.2, 119.4, 51.6, and 38.9; **MS** m/e 252 (M^+^).

### 4.16. Methyl 3-[2-(3-Butenyl)phenyl]acrylate (***13g***)

To a suspension of InCl_3_ (0.008 g, 0.04 mmol, 20 mol%) and 4Å molecular sieves (ca. 0.4 g), CH_2_Cl_2_ (6 mL) was added to allyltrimethylsilane (0.15 mL, 0.94 mmol, 5 equiv.) and **10** (0.0455 g, 0.179 mmol) at room temperature. The reaction was heated to reflux, stirred under N_2_ and monitored by TLC for 19 h. Following removal of volatiles under reduced pressure and purification by flash chromatography (5:1 PE:Et_2_O), **13g** was isolated as a light beige oil (0.0246 g, 64%, 78% BRMS). Continued elution afforded starting **10** (0.0083 g, 18%) in subsequent fractions. **IR** (neat) λ_max_ 3066, 2948, 1715, 1169, 979, and 763 cm^−1^; **^1^H NMR** (300 MHz, CDCl_3_) δ 8.03 (d, *J* = 15.9 Hz, 1H), 7.57 (d, *J* = 7.8 Hz, 1H), 7.32 (m, 1H), 7.18-7.27 (m, 2H), 6.38 (d, *J* = 15.9 Hz, 1H), 5.87 (m, 1H), 4.97–5.11 (m, 2H), 3.83 (s, 3H), 2.86 (dd, *J* = 9.7, 6.0 Hz, 2H), and 2.34 (m, 2H); **^13^C NMR** (75 MHz, CDCl_3_) δ 167.4, 142.3, 141.4, 137.4, 132.9, 130.0, 126.6, 126.5, 119.1, 115.4, 51.7, 35.4, and 32.7; the **HRMS** m/e for C_14_H_16_O_2_ calculated (M + 1)^+^ 217.1228, and found 217.1230.

### 4.17. Methyl 3-(2-(2-Methyl-3-oxo-3-phenylpropylphenyl)acrylate (***13h***)

To a suspension of InCl_3_ (0.0065 g, 0.029 mmol, 18 mol%) and 4Å molecular sieves (ca. 0.4 g) in 1,2-dichloroethane (5 mL) were added propiophenone trimethylsilyl enol ether (0.229 g, 1.11 mmol, 6.7 equiv.) and **10** (0.0422 g, 0.165 mmol) at room temperature. The reaction was heated to reflux, stirred under N_2_ and monitored by TLC for 15 h. Following a conventional extractive (CH_2_Cl_2_) workup and purification by preparative TLC (3:1 hexane:Et_2_O), **13h** was isolated as a light beige oil (0.0420 g, 82%). **IR** (neat) λ_max_ 3061, 2950, 1717, 1681, 1632, and 1597 cm^−1^; **^1^H NMR** (300 MHz, CDCl_3_) δ 8.10 (d, *J* = 15.8 Hz, 1H), 7.88 (m, 2H), 7.55 (m, 2H), 7.45 (m, 2H), 7.17–7.31 (m, 3H), 6.40 (d, *J* = 15.8 Hz, 1H), 3.84 (s, 3H), 3.72 (m, 1H), 3.30 (dd, *J* = 14.0, 6.6 Hz, 1H), 1.87 (dd, *J* = 14.0, 7.8 Hz), and 1.19 (d, *J* = 6.9 Hz, 3H); **^13^C NMR** (75 MHz, CDCl_3_) δ203.4, 167.2, 142.2, 139.4, 136.3, 133.3, 133.0, 131.2, 130.0, 128.7, 128.2, 127.0, 119.6, 51.7, 42.1, 36.6, and 17.3; **MS** m/e 508 (M^+^).

## Figures and Tables

**Table 1:** Ionization energies of select allyl- and dienyl bromides ^1^.

Molecule	E Ionization (a.u.)	E Ionization (kcal/mol)	E (rel) (kcal/mol)
7-Br	0.7034	441.4	0
8a-Br	0.6766	424.6	−16.8
8b-Br	0.6861	430.5	−10.9
8c-Br	0.6832	428.7	−12.7
9-Br	0.7144	448.3	+6.9

^1^ Calculations at the B3LYP 6-311++G(d,p)+ ZPVE level, in CH_2_Cl_2_.

**Table 2:** Optimization of **12a** formation.

Entry	Lewis Acid	T	Yield 12a (%)
1	CuCl (10 mol%)	rt	0
2	BiI_3_ (10 mol%)	rt	11
3	SnCl_4_ (10 mol%)	rt	36
4	SnCl_4_ (10 mol%)	40 °C	51
5	InCl_3_ (10 mol%)	rt	43
6	InCl_3_ (10 mol%)	40 °C	53
7	GaCl_3_ (10 mol%)	rt	68
8	GaCl_3_ (10 mol%)	40 °C	63
9	GaCl_3_ (5 mol%)	rt	47
10	GaCl_3_ (15 mol%)	rt	67
11	GaCl_3_ (10 mol%) ^1^	rt	51 (58 brsm) ^2^

^1^ Reaction conducted in the absence of 4 Å sieves. ^2^ brsm = based on recovered starting material.

**Table 3:** Results for condensation reactions of **8b**, **8c**.

Entry	Substrate	Nucleophile	Catalyst (mol%)	Time (h)	Product	Yield (%)
1	**8b**	mesitylene ^1^	GaCl_3_, 10	26	**12a**	68
2	**8b**	*p*-xylene ^1^	GaCl_3_, 10	24	**12b**	33 (54) ^2^
3	**8b**	*p*-xylene	GaCl_3_, 10	23	**12b**	65
4	**8b**	1,3-dimethoxybenzene	GaCl_3_, 10	23	**12c**	56
5	**8b**	1,3,5-trimethoxybenzene	GaCl_3_, 20	24	**12d**	51
6	**8b**	thiophene	GaCl_3_, 10	23	**12e/12e′**	63 (72:28) ^3^
7	**8b**	allyltrimethylsilane	InCl_3_, 10	24	**12f**	53
8	**8b**	allyltrimethylsilane	InCl_3_, 20	14	**12f**	66
9	**8c**	mesitylene	GaCl_3_, 10	20	**12g**	50

^1^ Reaction conducted at room temperature. ^2^ Yield based on recovered SM. ^3^ **12**:**12′** ratio.

**Table 4:** Results for reactions of **10**.

Entry	Substrate	Nucleophile	Catalyst (mol%)	Time (h)	Product	Yield (%)
1	**10**	mesitylene	GaCl_3_, 10	24	**13a**	73
2	**10**	*p*-xylene	GaCl_3_, 10	21	**13b**	76
3	**10**	1,3-diimethoxybenzene	GaCl_3_, 10	22	**13c**	77
4	**10**	1,3,5-trimethoxybenzene	GaCl_3_, 20	22	**13d**	75
5	**10**	thiophene	GaCl_3_, 10	20	**13e/13e′**	92 (71:29) ^1^
6	**10**	benzene	GaCl_3_, 30	30	**13f**	72
7	**10**	allyltrimethylsilane	GaCl_3_, 10	24	**13g**	0
8	**10**	allyltrimethylsilane	GaCl_3_, 50	24	**13g**	46
9	**10**	Allyltrimethylsilane ^2^	InCl_3_, 10	24	**13g**	29
10	**10**	Allyltrimethylsilane ^2^	InCl_3_, 20	19	**13g**	64 (78) ^2^
11	**10**	propiophenone TMS enol ether ^3^	InCl_3_, 20	15	**13h**	82

^1^ **13**:**13′** ratio. ^2^ Yield based on recovered SM. ^3^ Reaction conducted in ClCH_2_CH_2_Cl at reflux.

## Data Availability

Not applicable.

## Supplementary Materials

The following supporting information can be downloaded at: https://www.mdpi.com/article/10.3390/molecules27103078/s1. Copies of the ^1^H NMR and ^13^C NMR spectra of all new compounds. Final coordinates for the computationally determined structures.

## Sample Availability

Samples of all compounds are available from the authors.
