# Supplementary material for: Generation and Reactions of ε-Carbonyl Cations via Group 13 Catalysis
Source: Molecules. 2022 May 11;27(10):3078. doi: 10.3390/molecules27103078 (PMC9146154; doi:10.3390/molecules27103078)
Supplement: Supplementary file 1 [file molecules-27-03078-s001.zip › molecules-1686125-supplementary.pdf]

Supporting Information for

Generation and Reactions of ε-Carbonyl Cations via Group 13 Catalysis

Page M. Penner and James R. Green*

Department of Chemistry and Biochemistry, University of Windsor, Windsor, ON, N9B 3P4, Canada

*Correspondence: jgreen@uwindsor.ca

^{1}H and ^{13}C NMR Spectra for Newly Prepared Compounds S2

^{1}H and ^{13}C NMR Spectra - **8c** S2
^{1}H and ^{13}C NMR Spectra – **10** S4
^{1}H and ^{13}C NMR Spectra - **12a** S6
^{1}H and ^{13}C NMR Spectra – **12b** S8
^{1}H and ^{13}C NMR Spectra – **12c** S10
^{1}H and ^{13}C NMR Spectra – **12d** S12
^{1}H and ^{13}C NMR Spectra – **12e/12e'** S14
^{1}H and ^{13}C NMR Spectra – **12f** S16
^{1}H and ^{13}C NMR Spectra – **12g** S18
^{1}H and ^{13}C NMR Spectra – **13a** S20
^{1}H and ^{13}C NMR Spectra – **13b** S22
^{1}H and ^{13}C NMR Spectra – **13c** S24
^{1}H and ^{13}C NMR Spectra – **13d** S26
^{1}H and ^{13}C NMR Spectra – **13e/13e'** S28
^{1}H and ^{13}C NMR Spectra – **13f** S30
^{1}H and ^{13}C NMR Spectra – **13g** S32
^{1}H and ^{13}C NMR Spectra – **13h** S34

Cartesian Coordinates of Calculationally Optimized Structures S36

pmp.098 30, 1H, Br-CH2-C=C-C=C-C(O)Ph, 500 MHz, 3/17/22

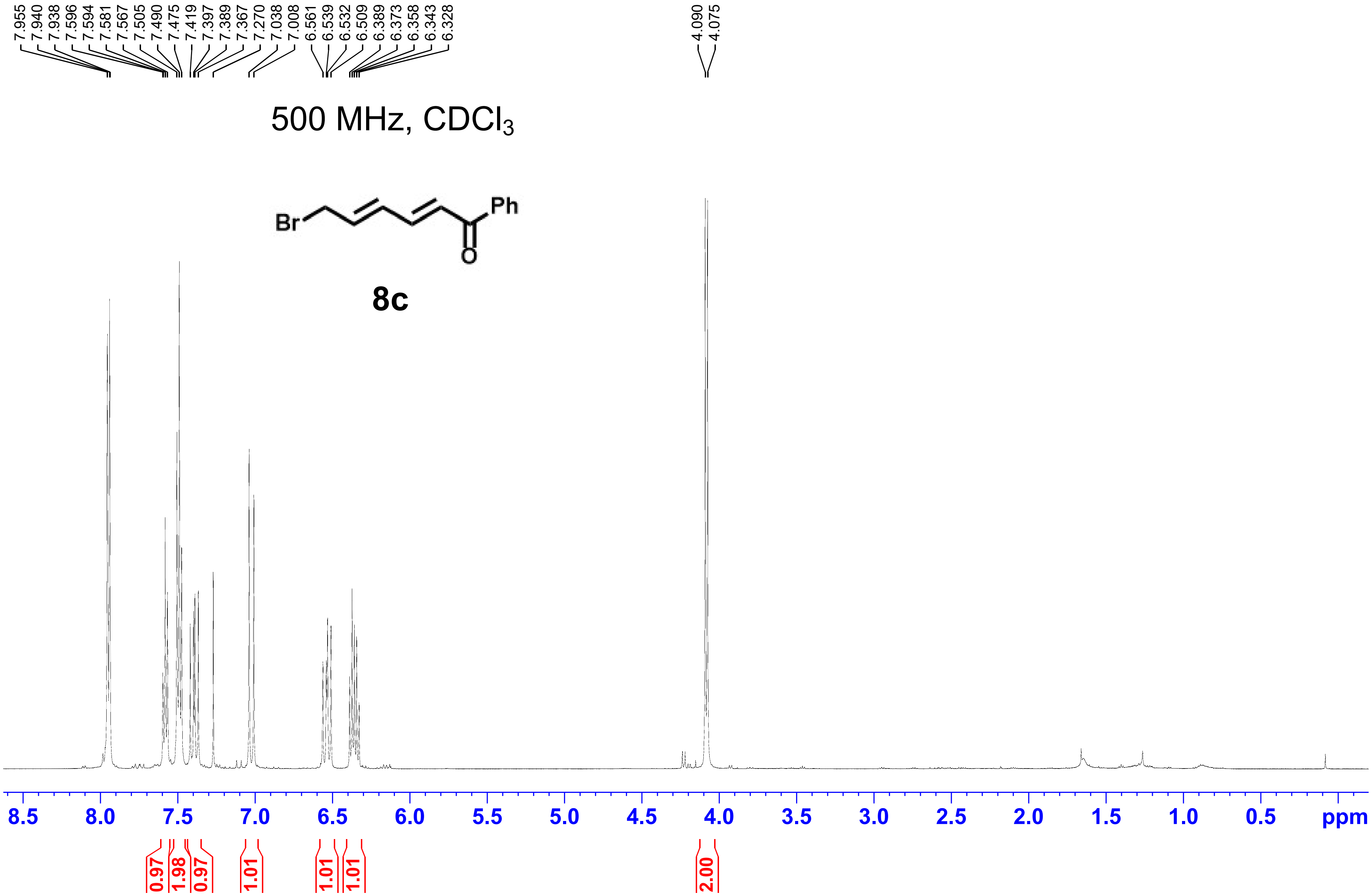

pmp.098 31, 13C, Br-CH2-C=C-C=C-C(O)Ph, 500/125 MHz, 3/17/22

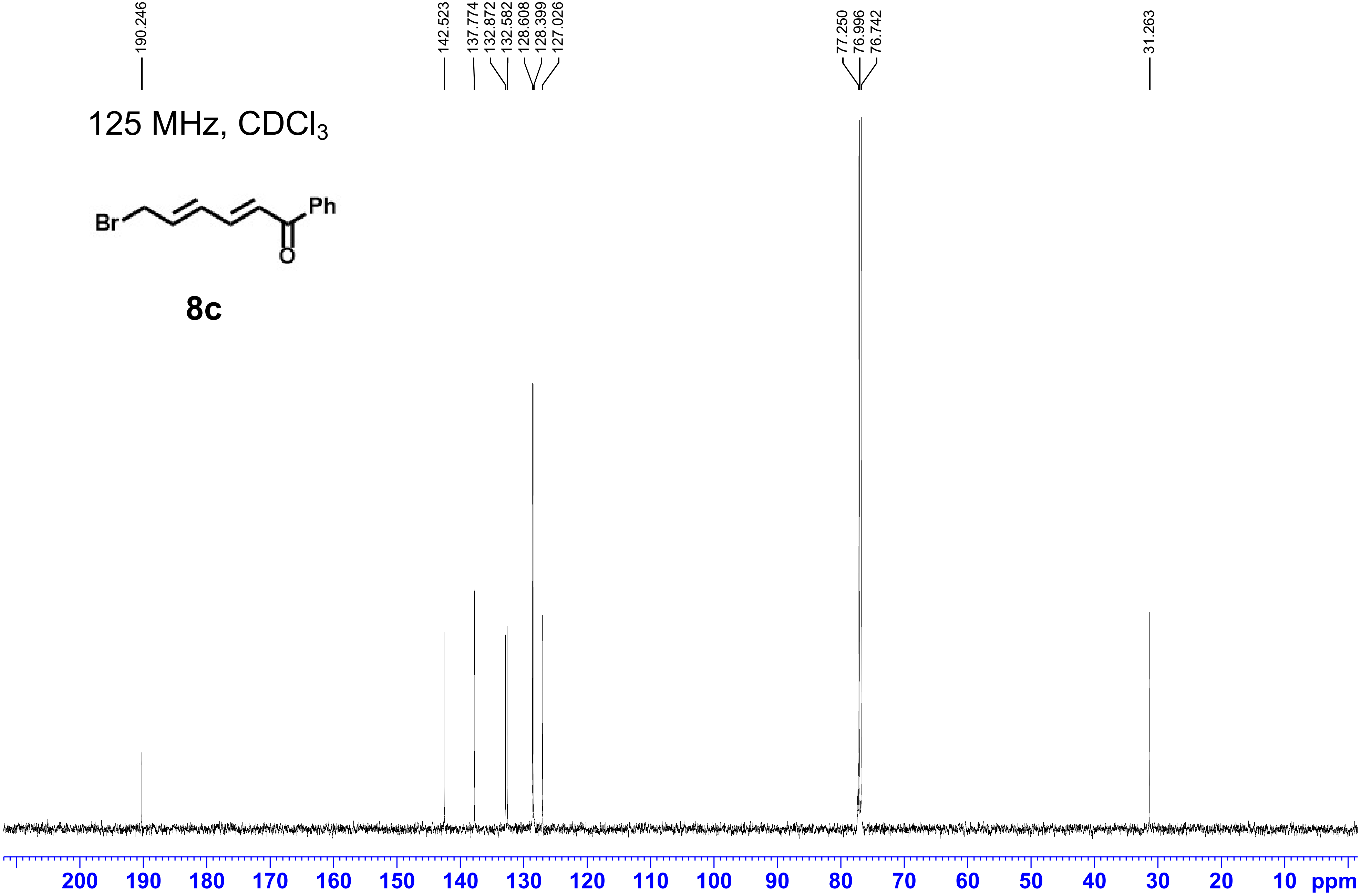

125 MHz, $CDCl_3$

8c

pmp.143b SM C
1D 1H 300 MHz B82

300 MHz, $CDCl_3$

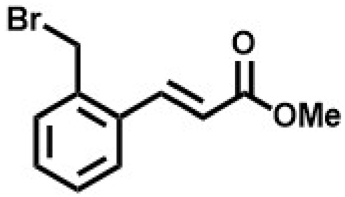

10

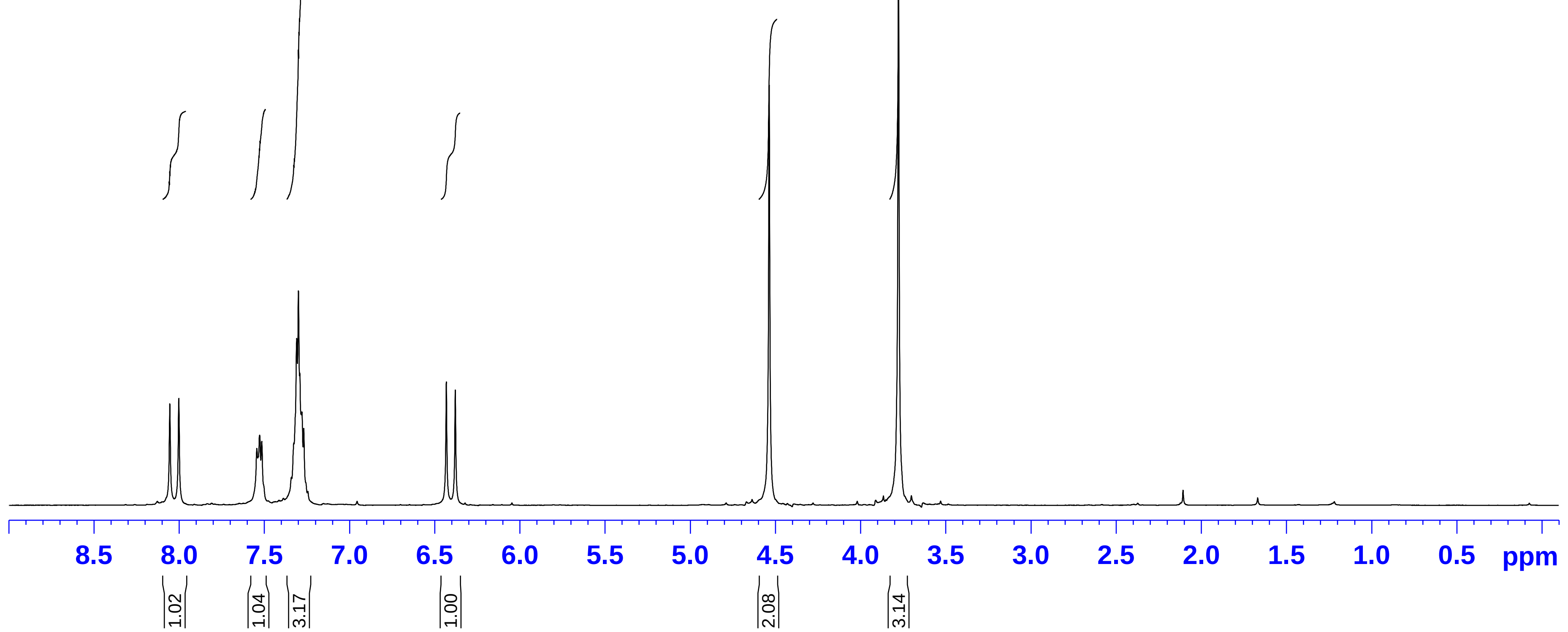

pmp.143bc 20 SM C
13C 1D 300 MHz B82

166.725
140.458
136.409
133.426
130.497
130.120
129.059
127.028
120.352
51.614
30.404

75 MHz, $CDCl_3$

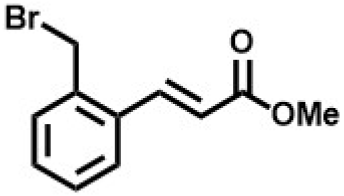

10

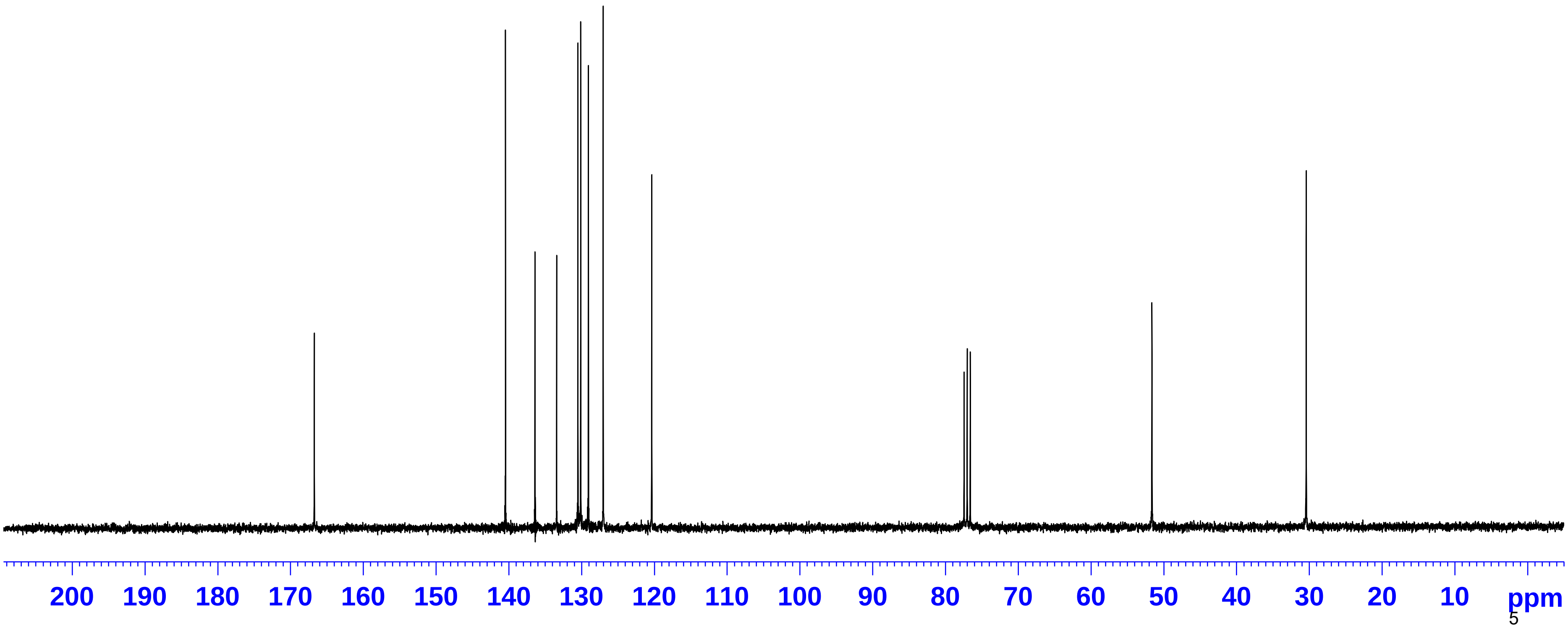

pmp.031 20, 1H, sorbate + mesitylene, 1H, 500, 1/15/22

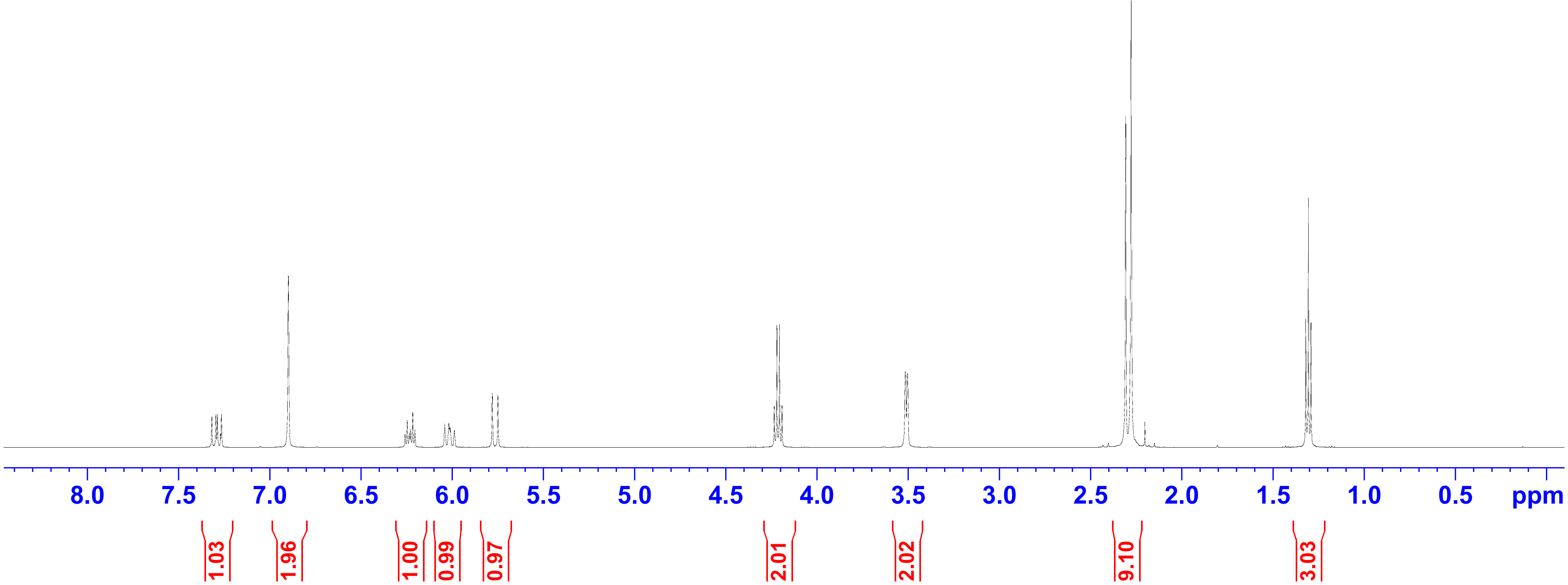

500 MHz, $CDCl_3$

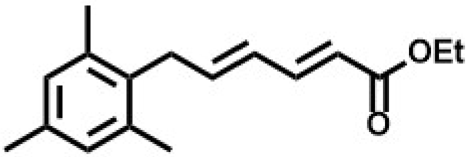

12a

pmp.031 21, 13C, sorbate + mesitylene adduct, 500/125, 1/15/22

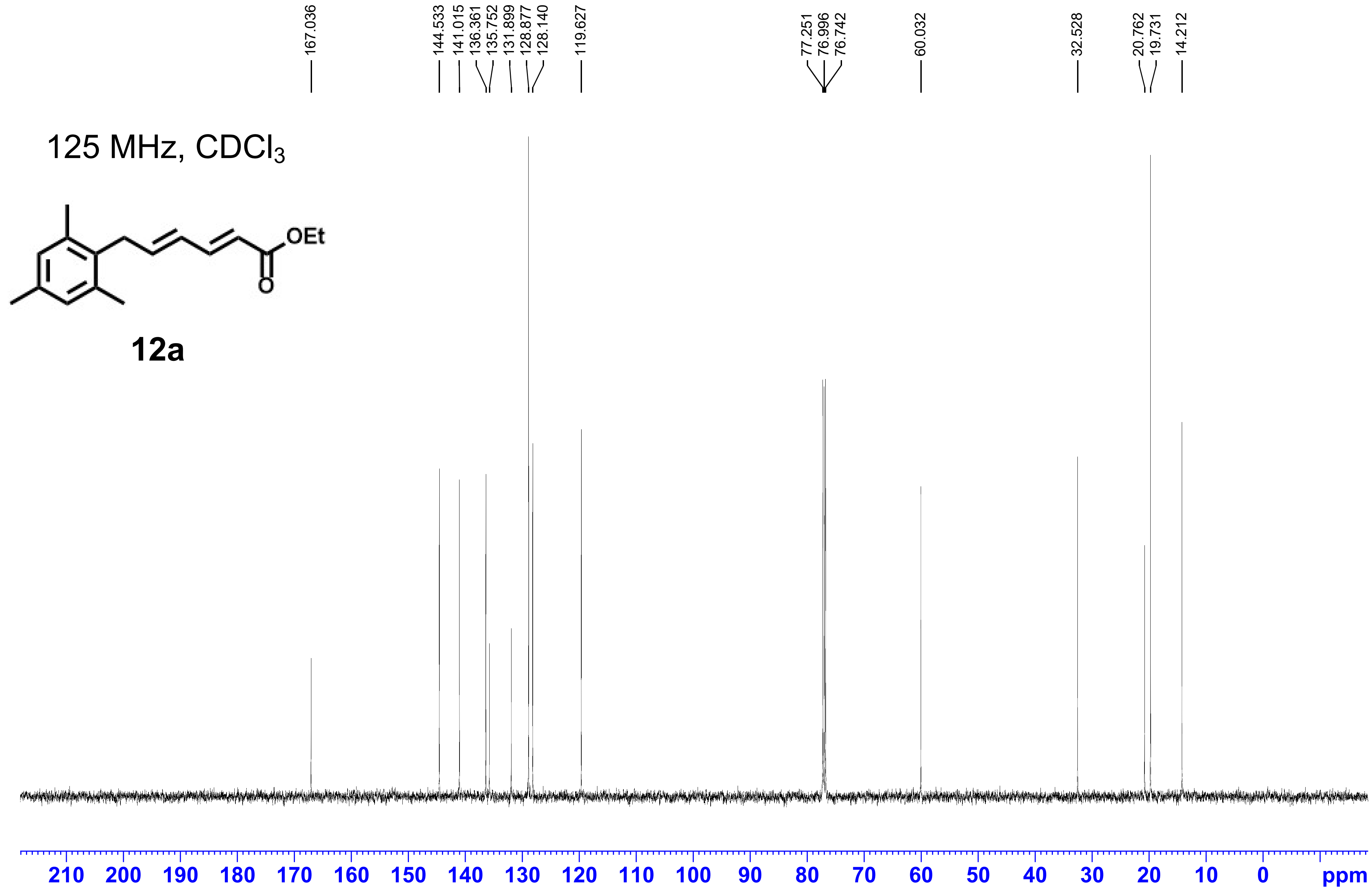

pmp.040 20, sorbate + p-xylene, 1H, 500, 1/24/22

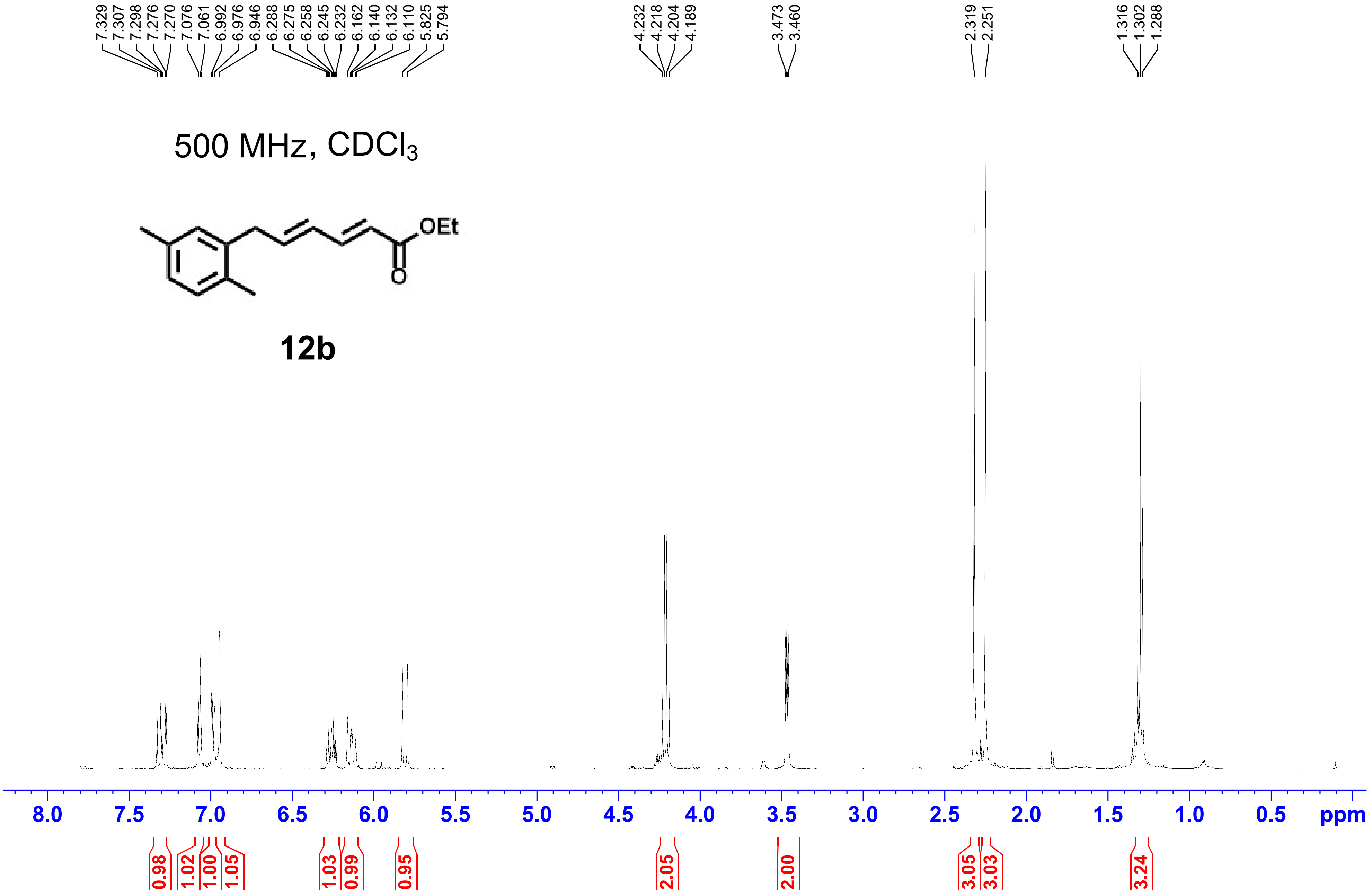

500 MHz, $CDCl_3$

12b

pmp.040 21, sorbate + p-xylene, 13C, 500/125, 1/24/22

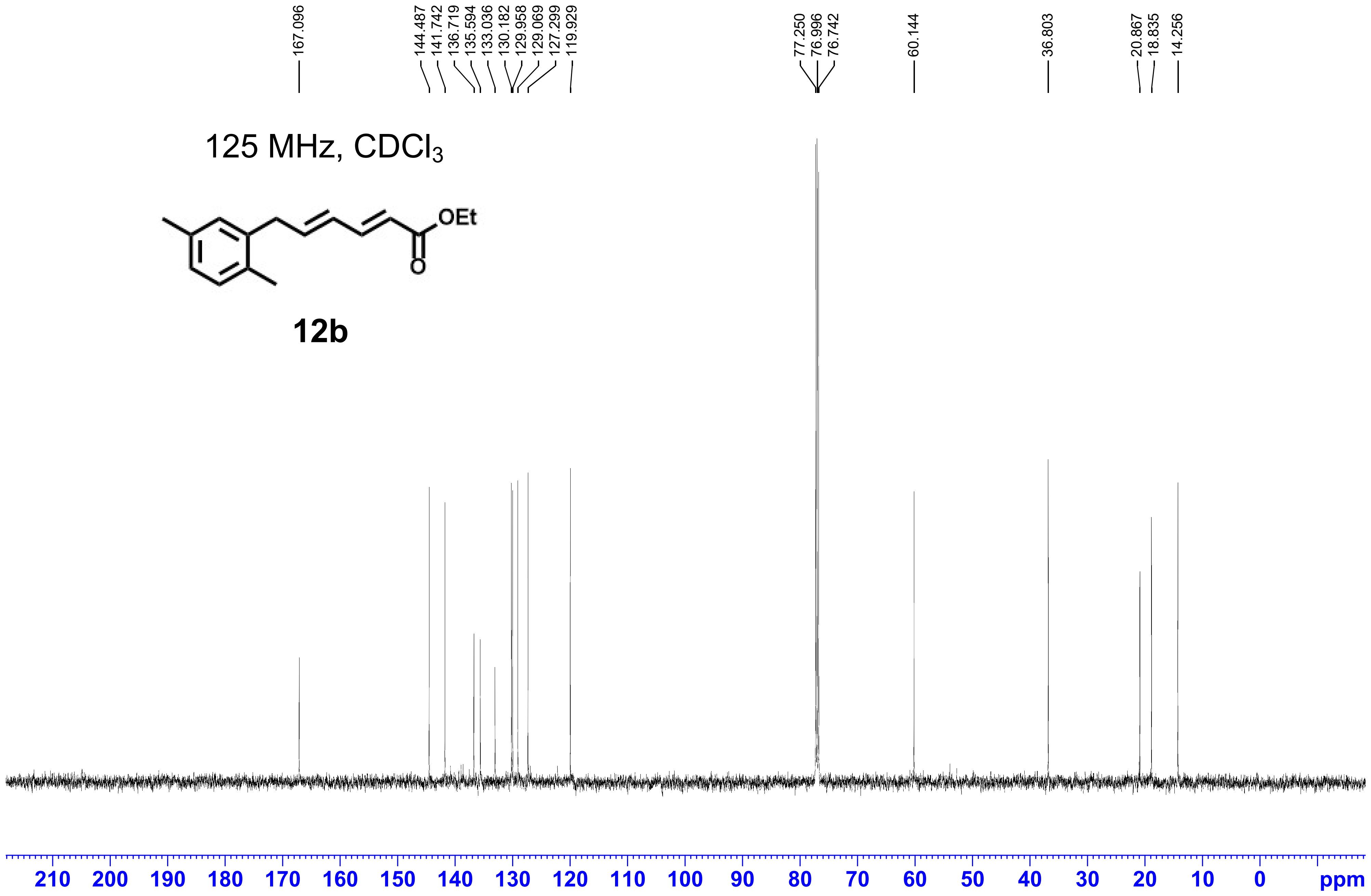

pmp.057 20, 1H, sorbate + 13-MeO2Ph, 1H, 500, 1/17/22

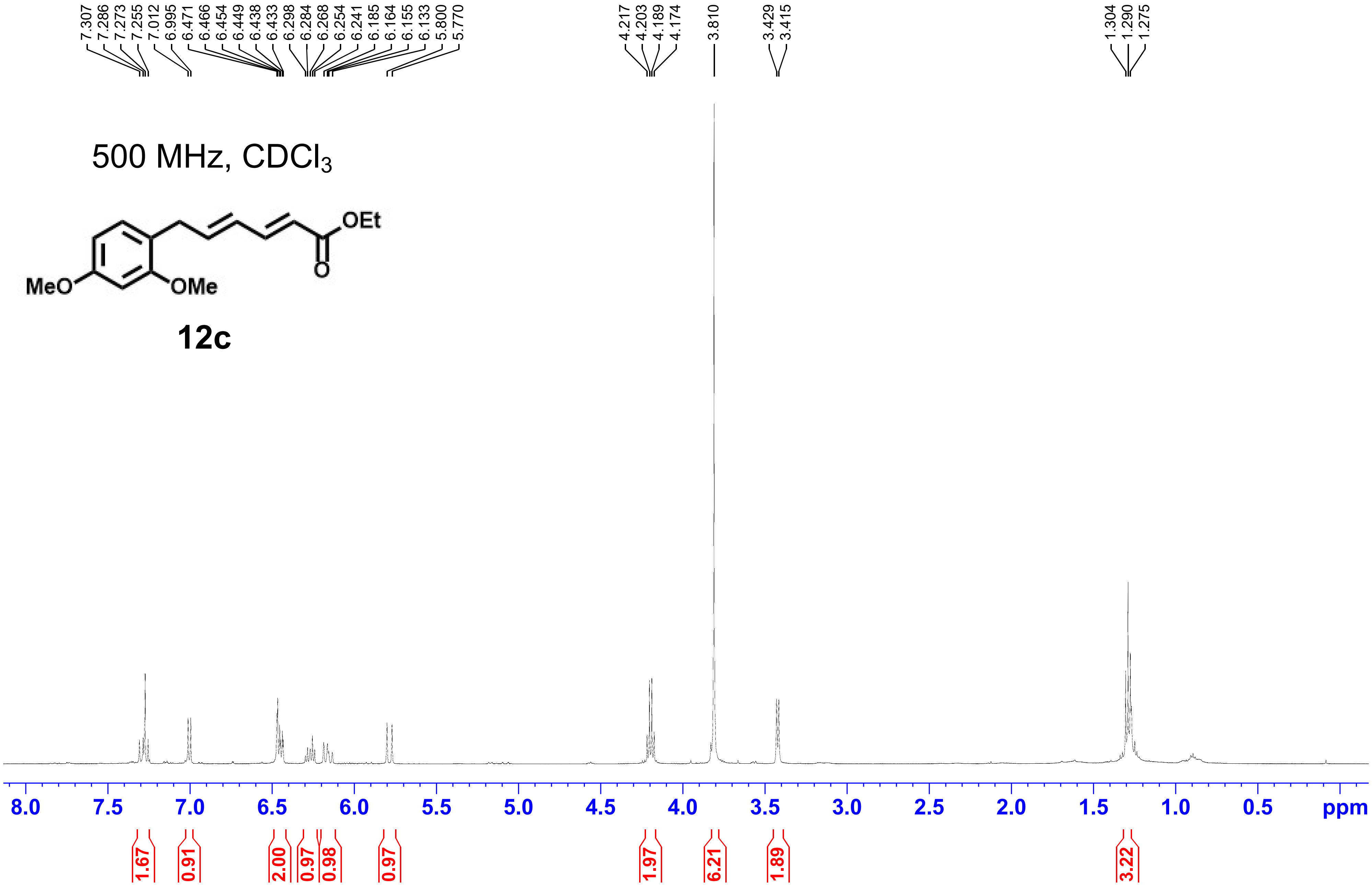

12c

pmp.057 21, 13C, sorbate + 1,3-MeO2Ph, 500/125, 1/17/22

125 MHz, $CDCl_3$

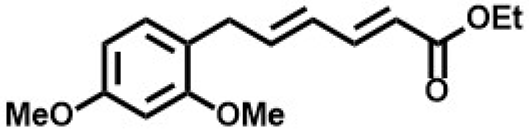

12c

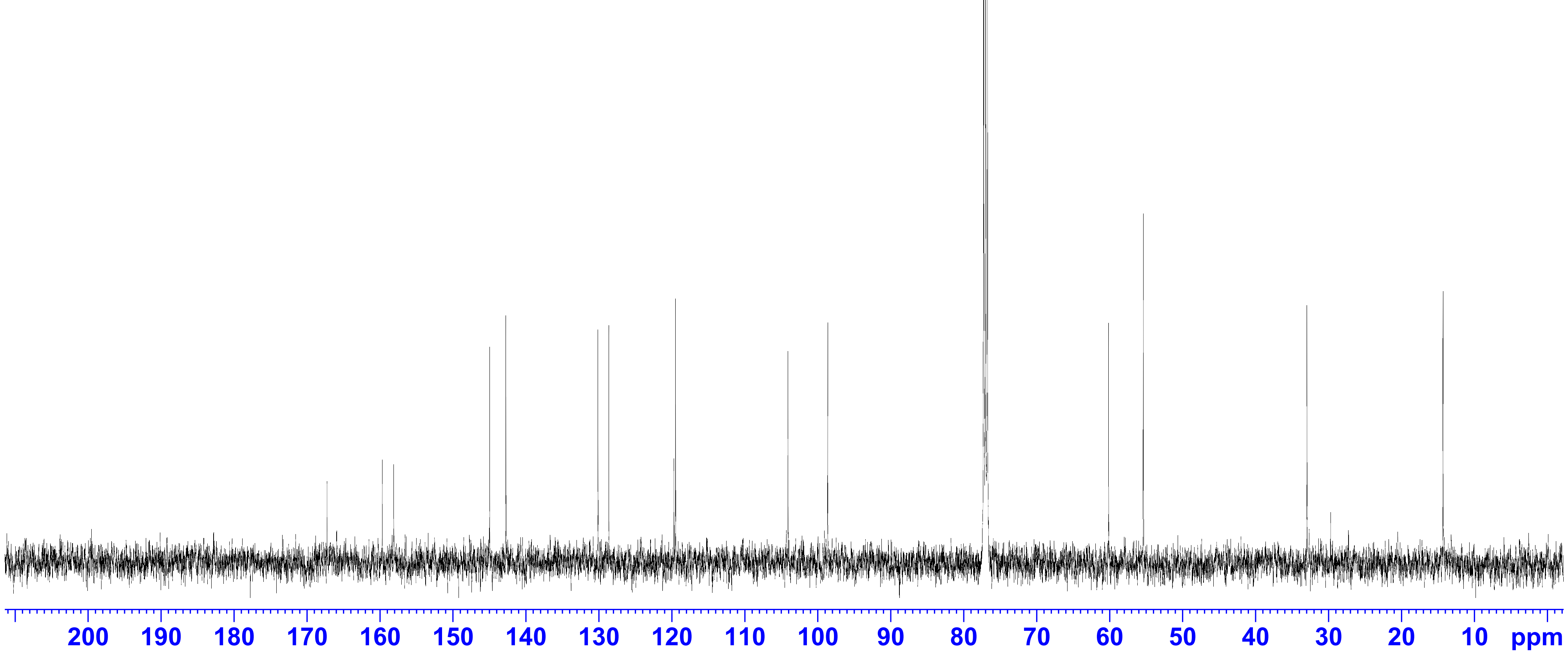

pmp.069 20, 1H, sorbate + 135-MeO3Ph, 1H, 500, 1/15/22

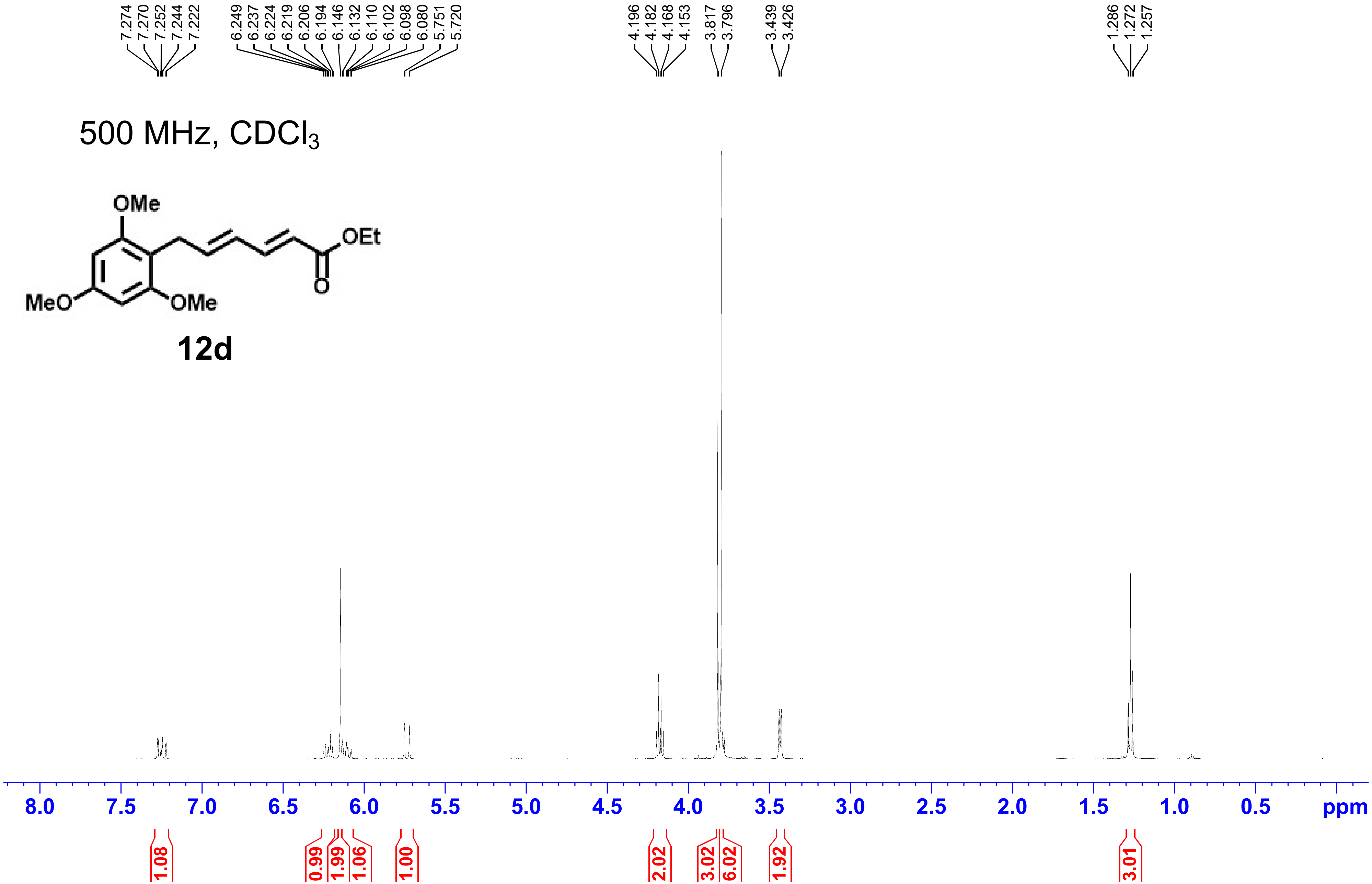

pmp.069 21, 13C, sorbate + 135-MeO3Ph, 500/125, 1/15/22

125 MHz, $CDCl_3$

12d

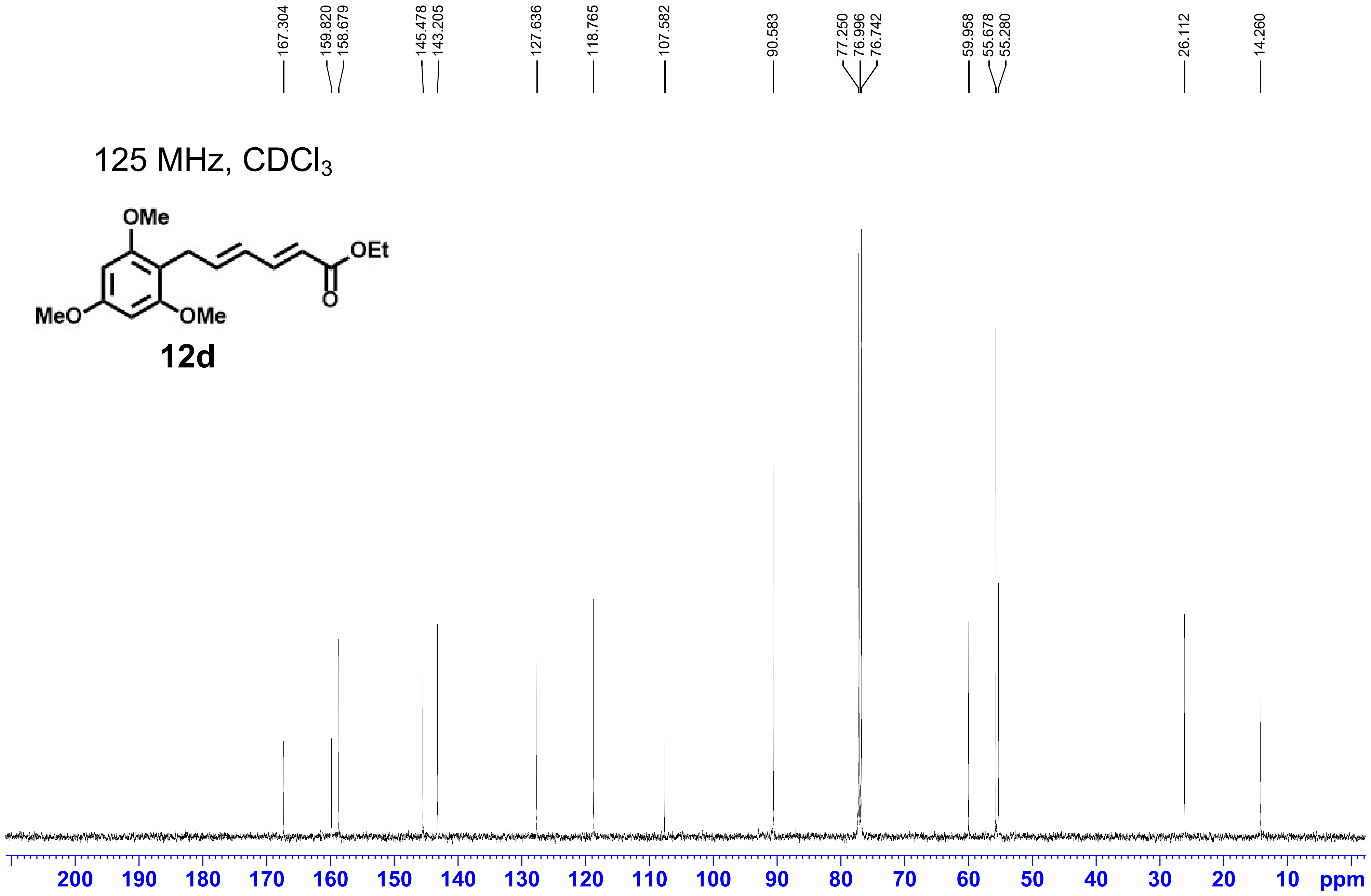

pmp.074 20, sorbate + thiophene, 1H, 500, 1/22/22

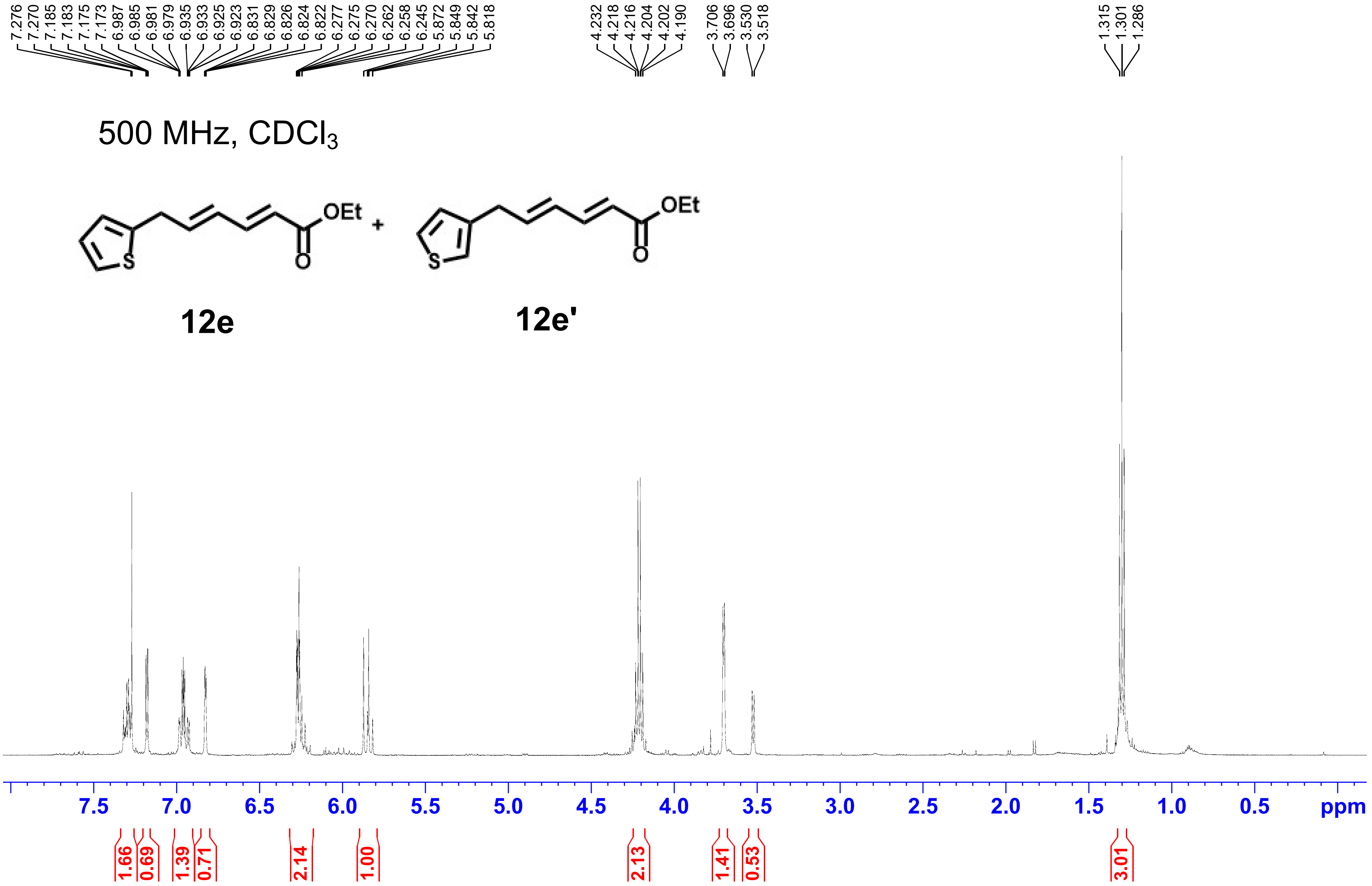

pmp.074 21, sorbate + thiophene, 13C, 500/125, 1/22/22

125 MHz, $CDCl_3$

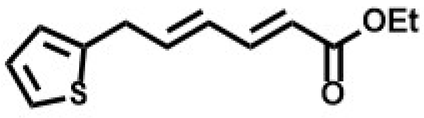

+

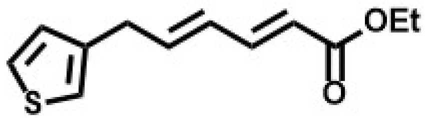

12e **12e'**

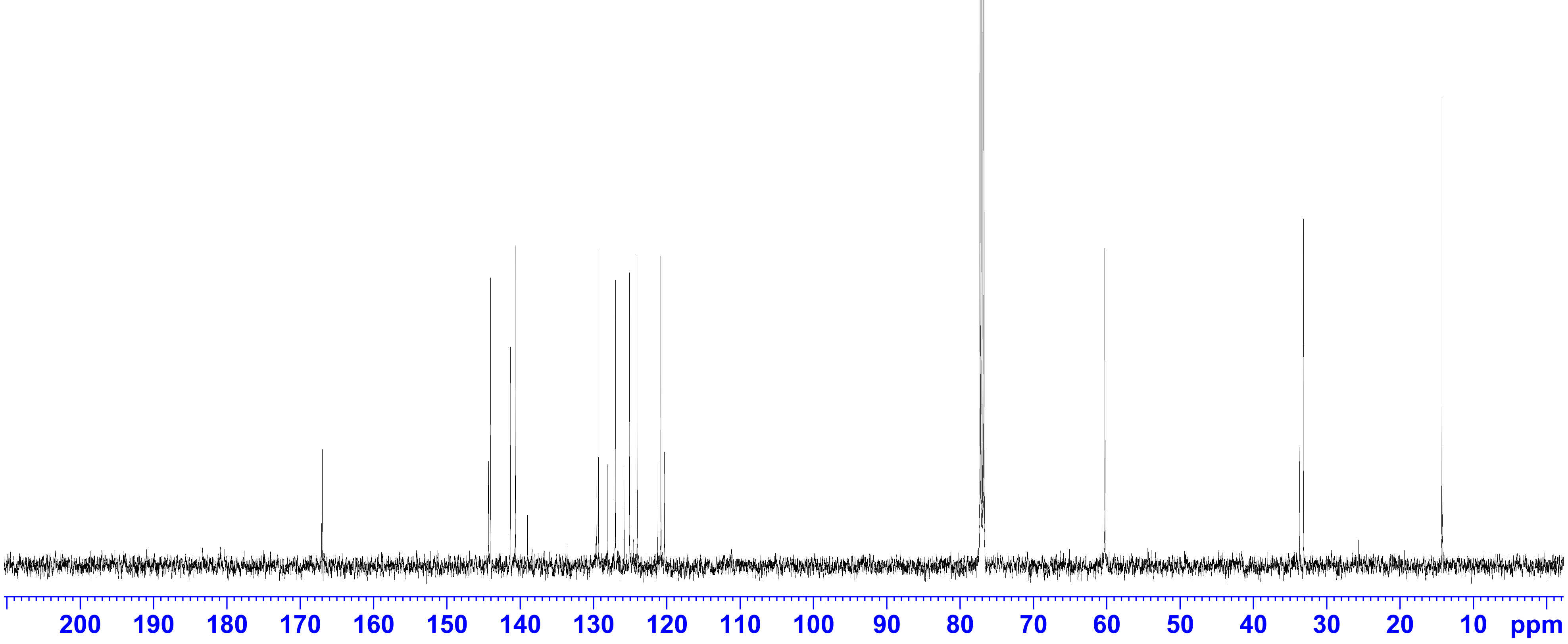

pmp.079 30, sorbate + allylTMS, 1H, 500, 3/20/22

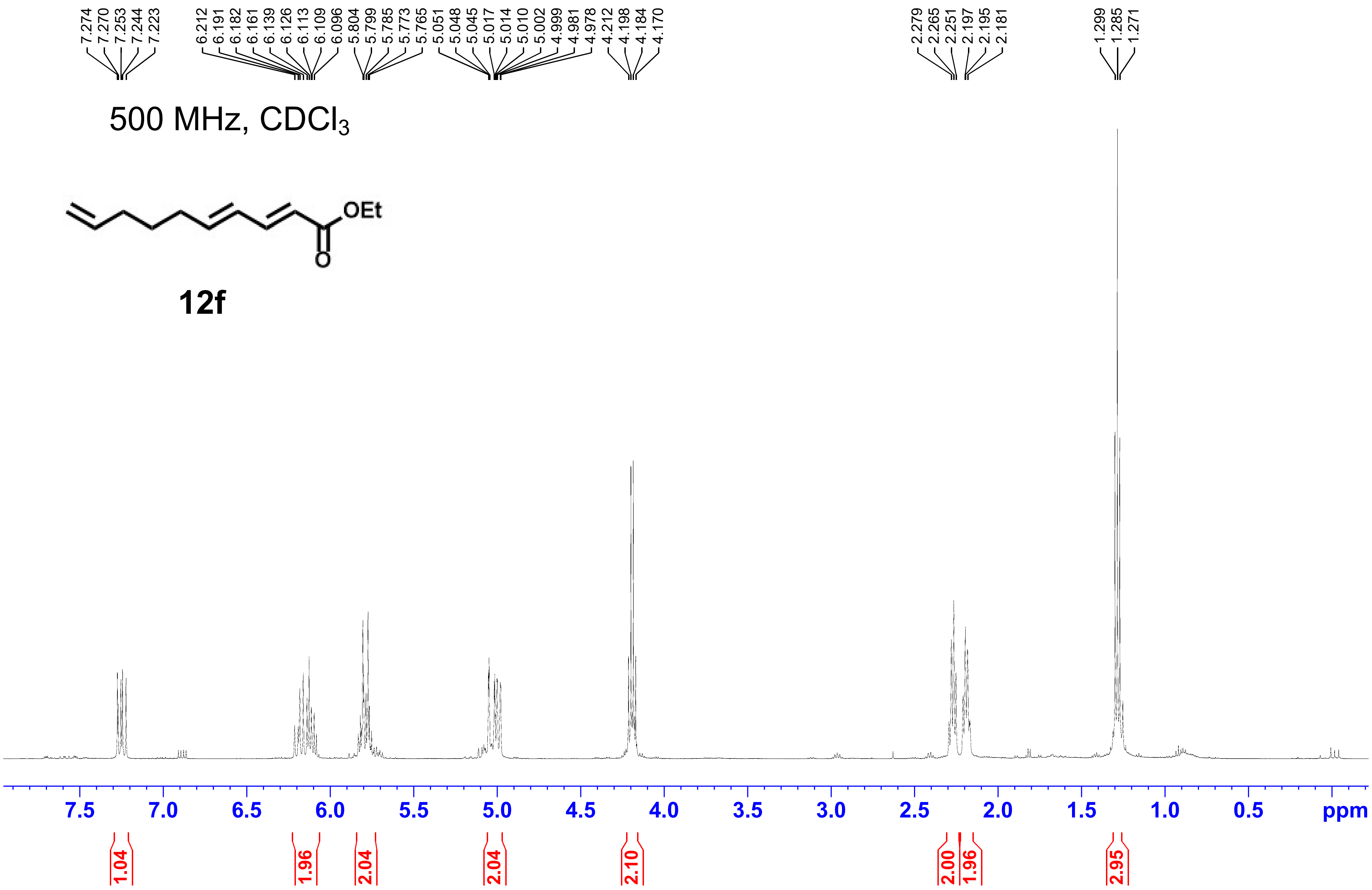

pmp.079 30, sorbate + allylTMS, 13C, 500/125, 3/20/22

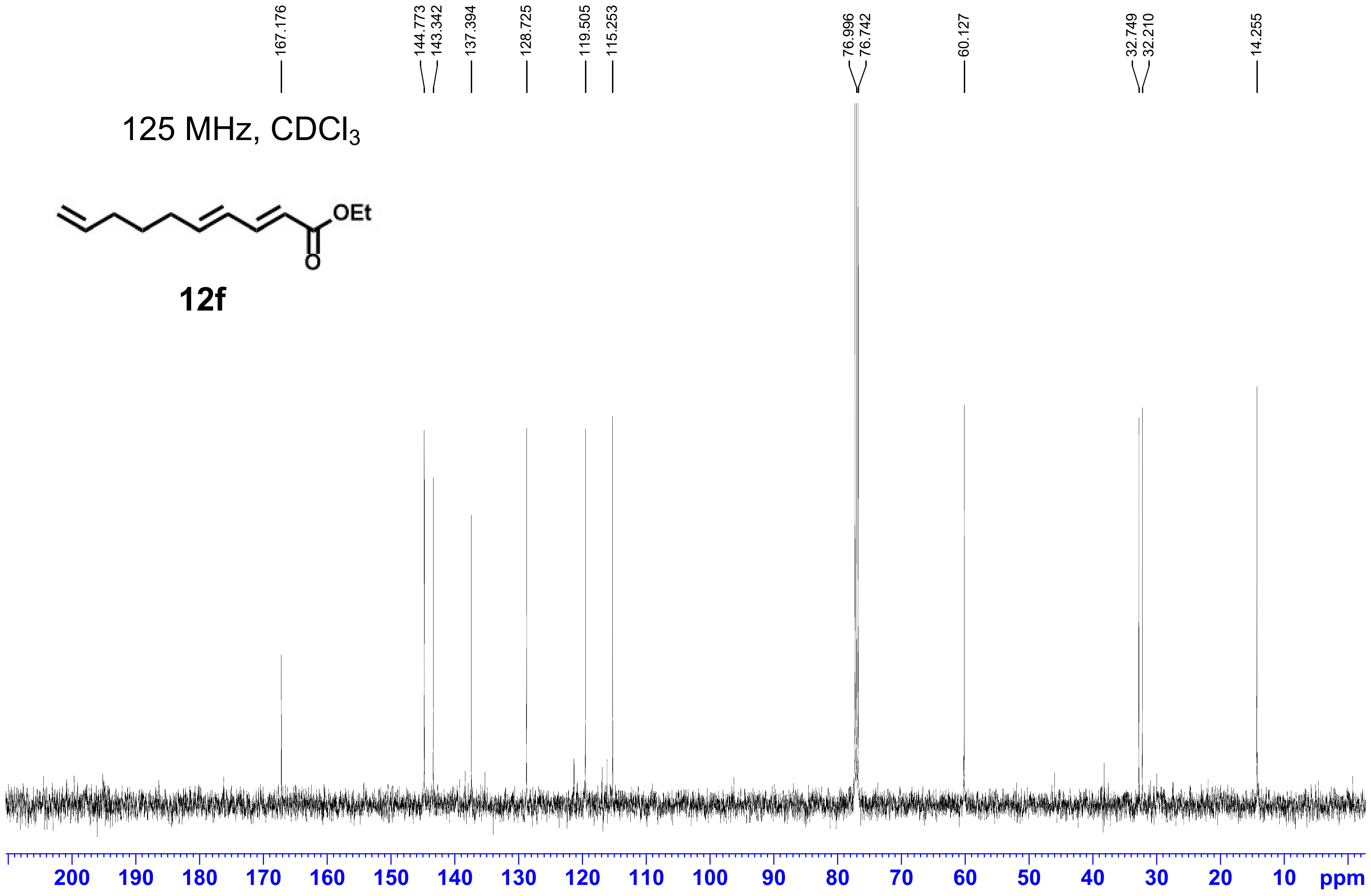

pmp.100 20, 1H, Ph ket + mesitylene, 300US, 2/4/22

300 MHz, $CDCl_3$

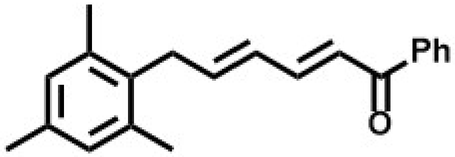

12g

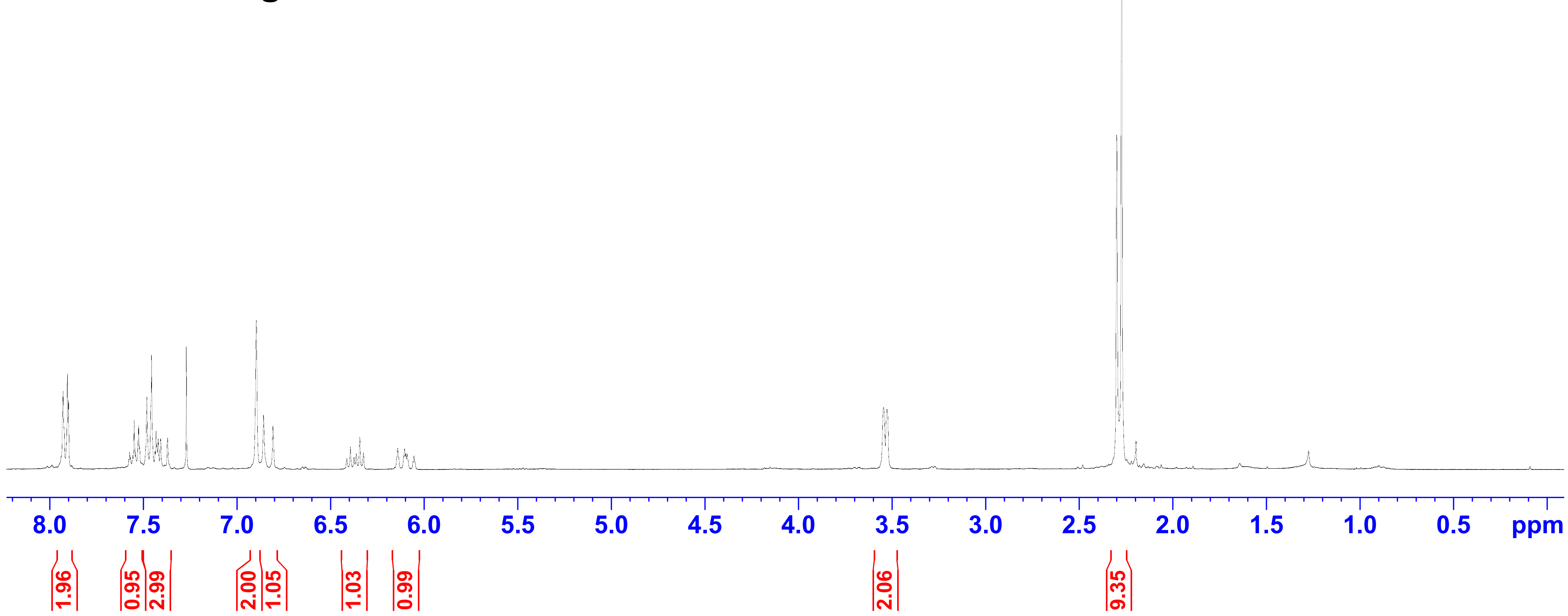

pmp.100 21, 13C, Ph ketone + mesitylene, 300US/75, 2/4/21

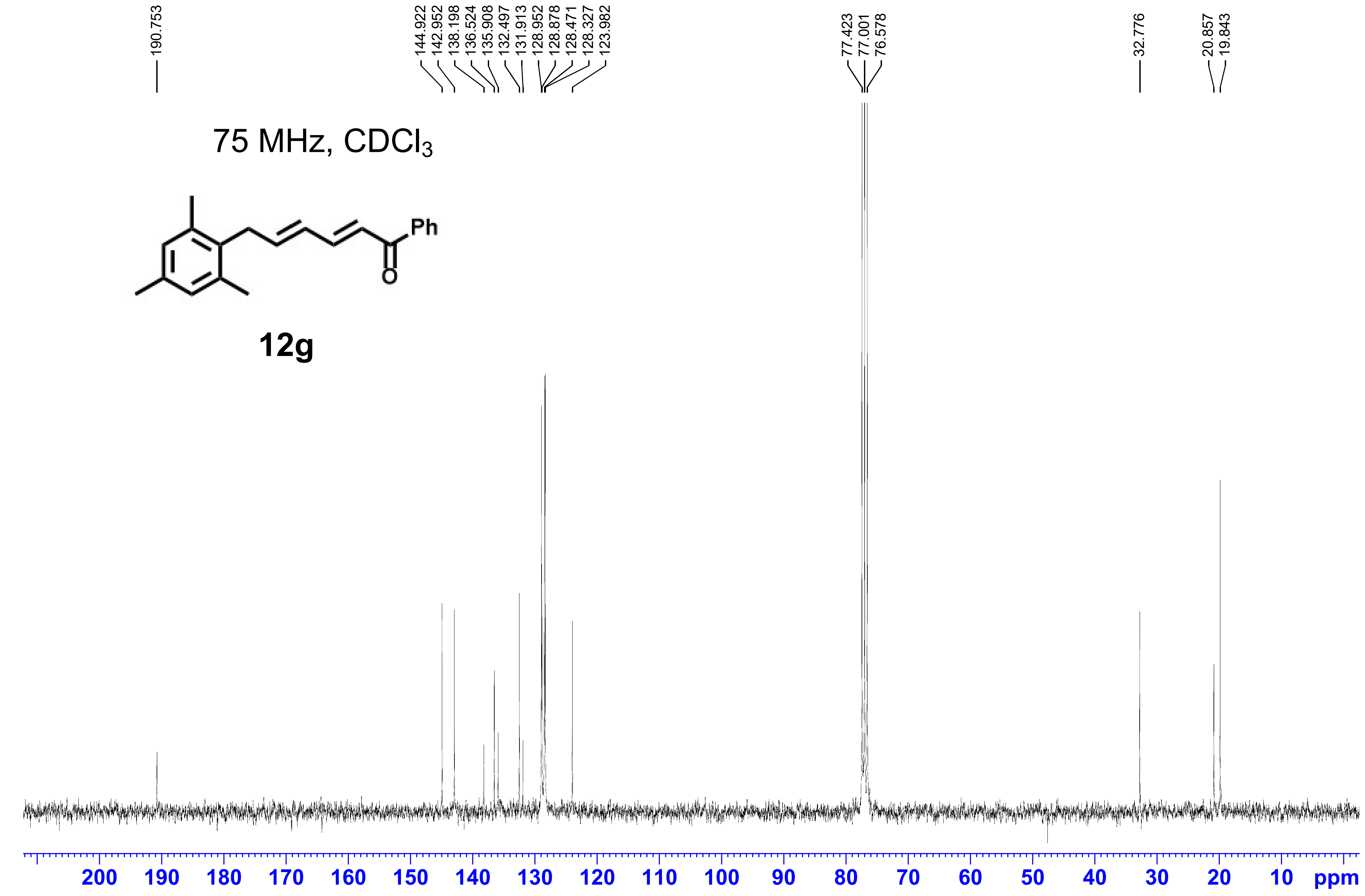

pmp.148 20, phenyl fused + mesitylene, 1H, 500, 1/20/22

500 MHz, $CDCl_3$

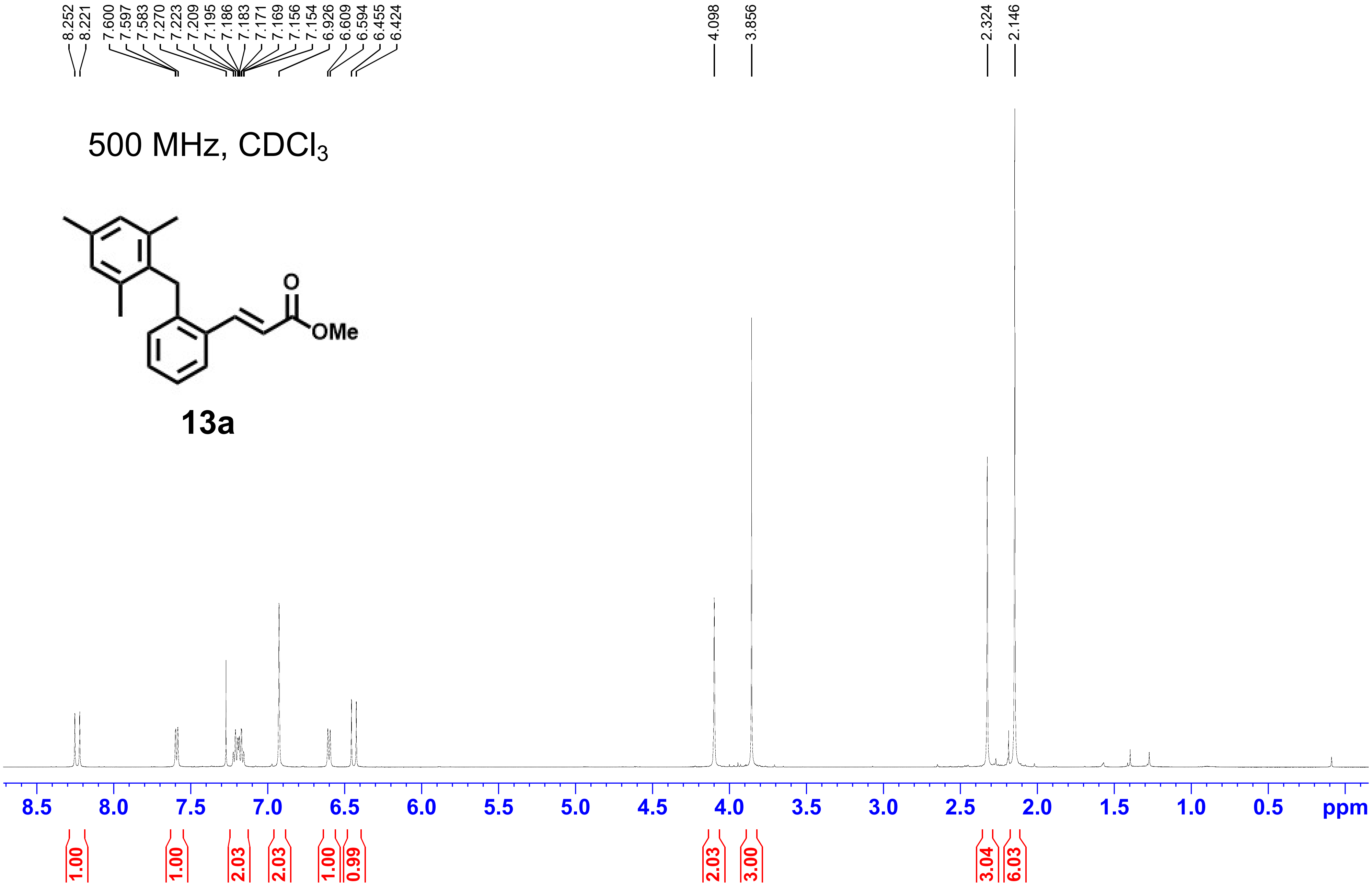

13a

pmp.148 21, phenyl fused + mesitylene, 13C, 500/125, 1/20/22

125 MHz, $CDCl_3$

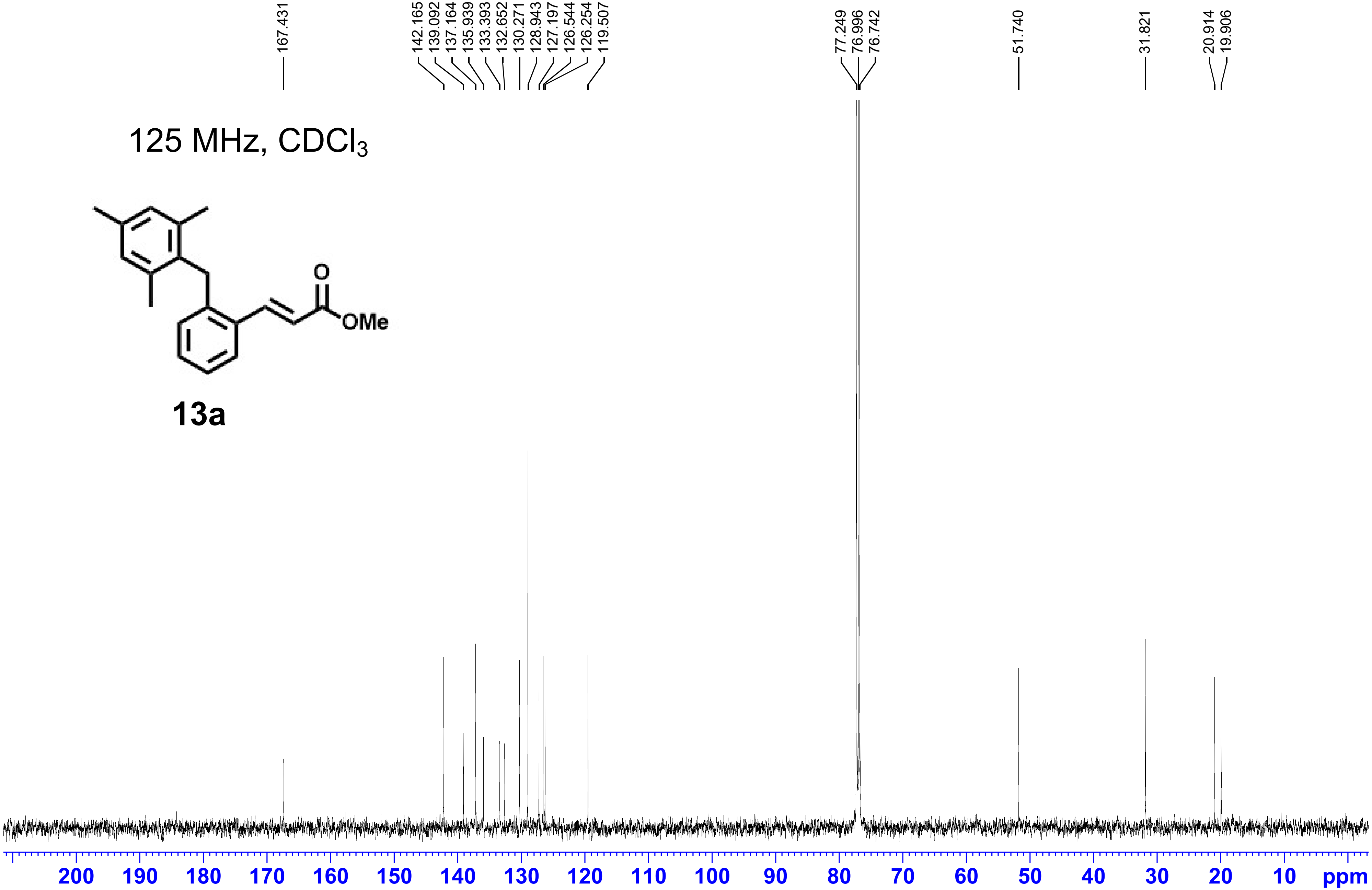

13a

pmp.150 20, phenyl fused + p-xylene, 1H, 500, 1/19/22

500 MHz, $CDCl_3$

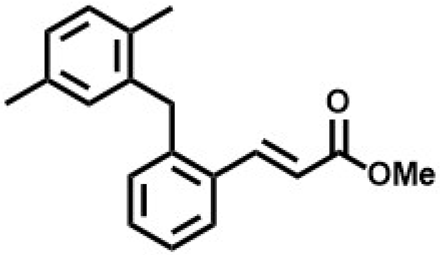

13b

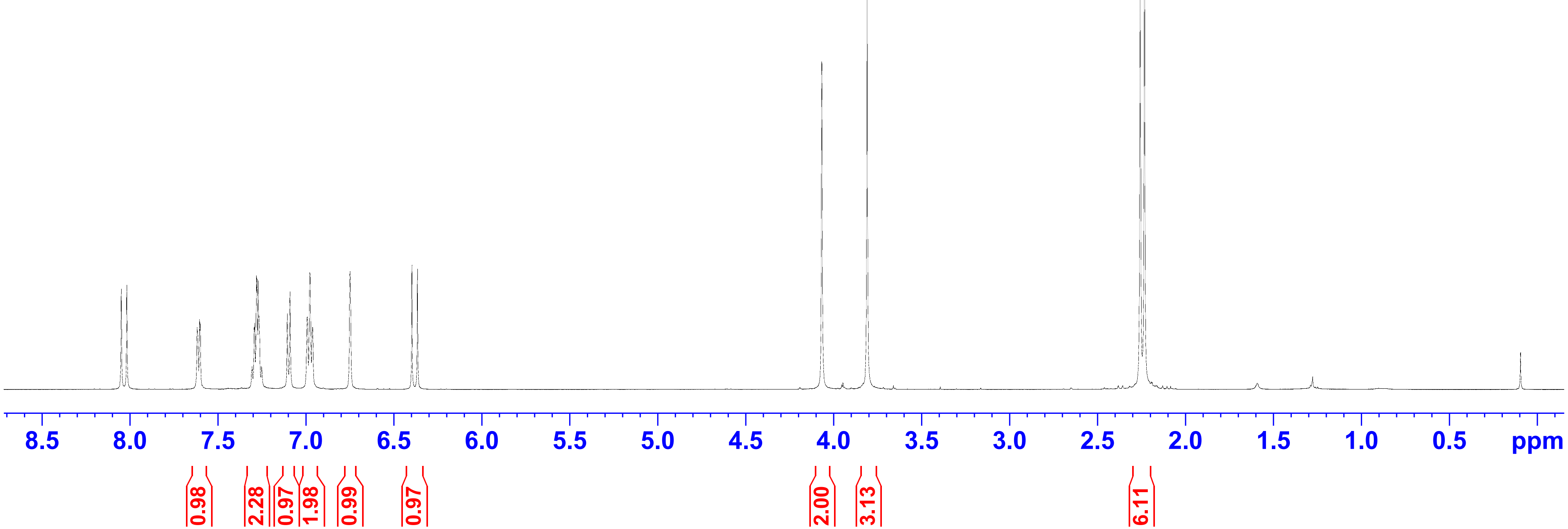

pmp.150 21, phenyl fused + p-xylene, 13C, 500/125, 1/19/22

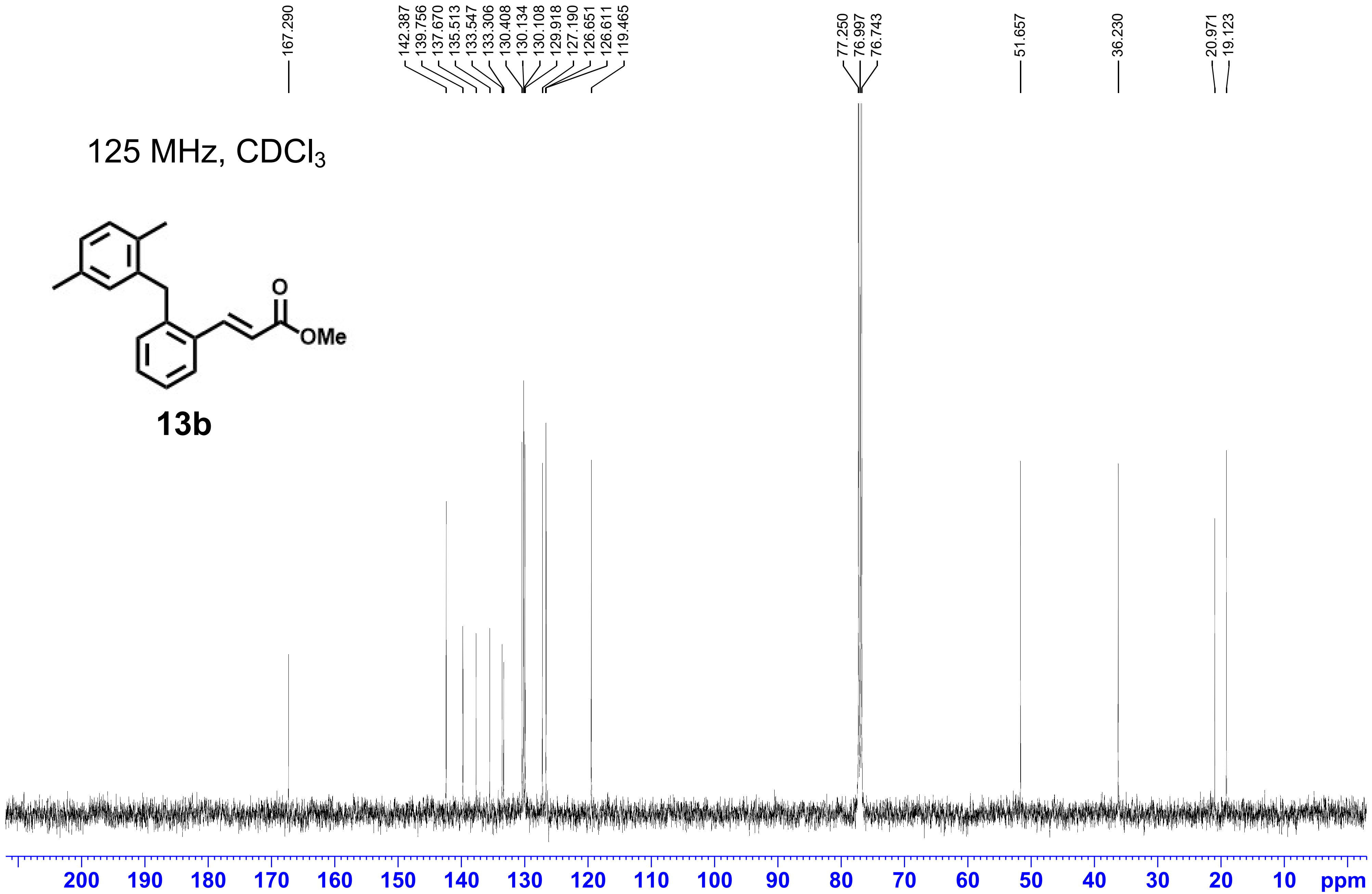

125 MHz, $CDCl_3$

13b

pmp.141 20, 1H, benzo + 1,3-MeO2Ph, B82-300, 1/26/22

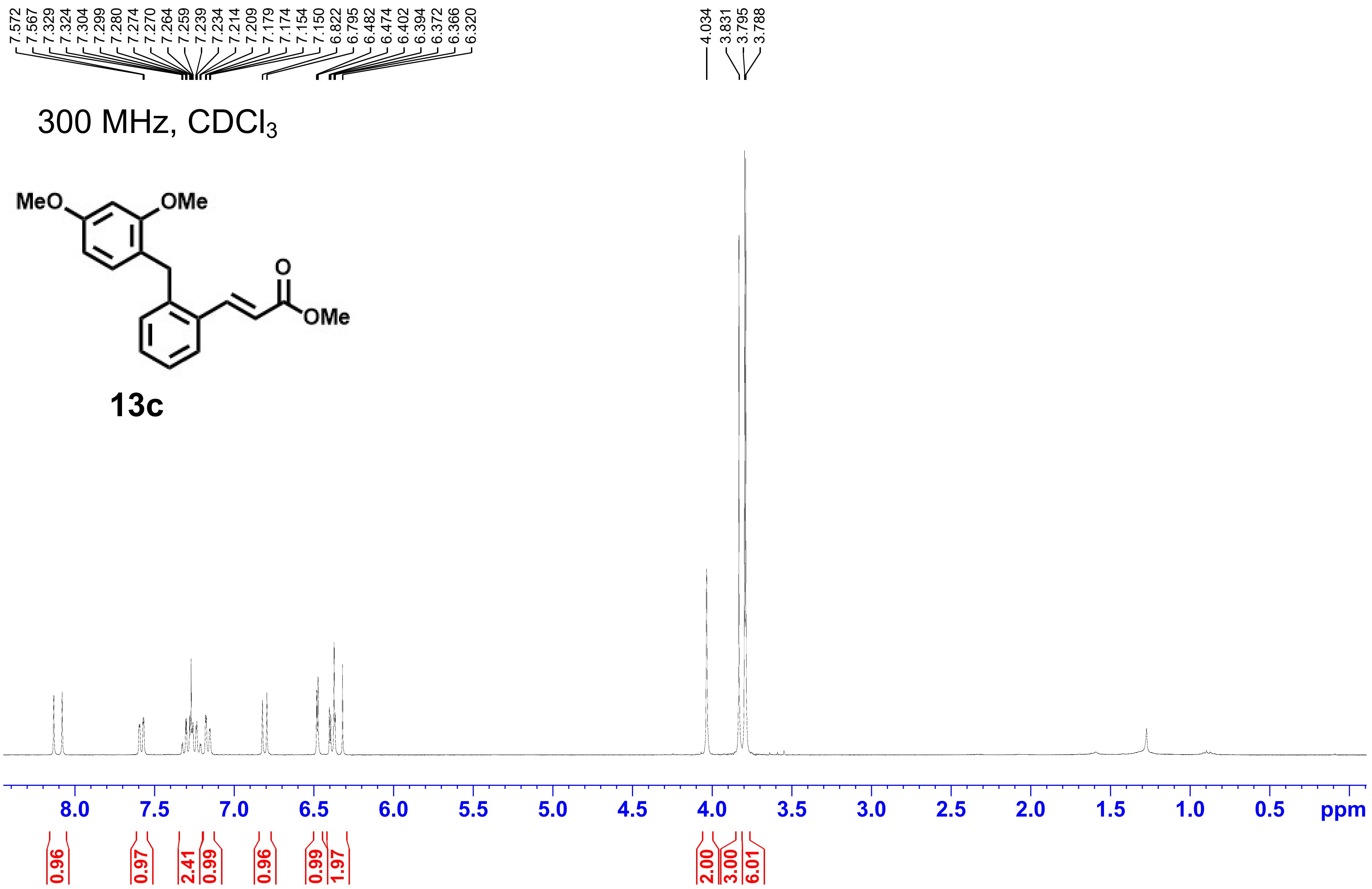

13c

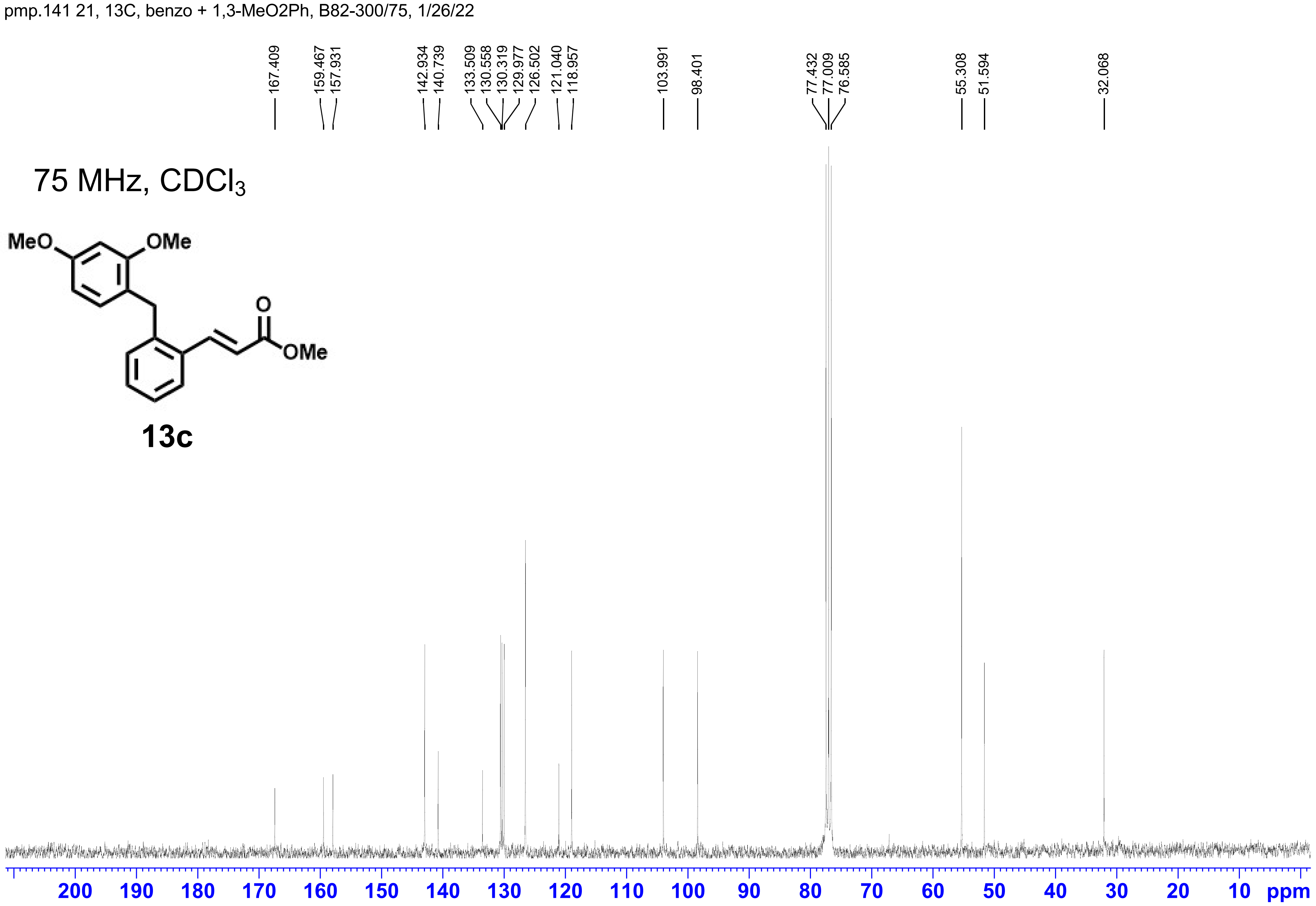
pmp.141 21, 13C, benzo + 1,3-MeO2Ph, B82-300/75, 1/26/22
167.409
159.467
157.931
142.934
140.739
133.509
130.558
130.319
129.977
126.502
121.040
118.957
103.991
98.401
77.432
77.009
76.585
55.308
51.594
32.068
75 MHz, CDCl3
MeO
OMe
O
OMe
200
190
180
170
160
150
140
130
120
110
100
90
80
70
60
50
40
30
20
10
ppm

13c

pmp.149 20, 1H, aryl fused substrate + 135-MeO3Ph, B82-300, 1/18/22

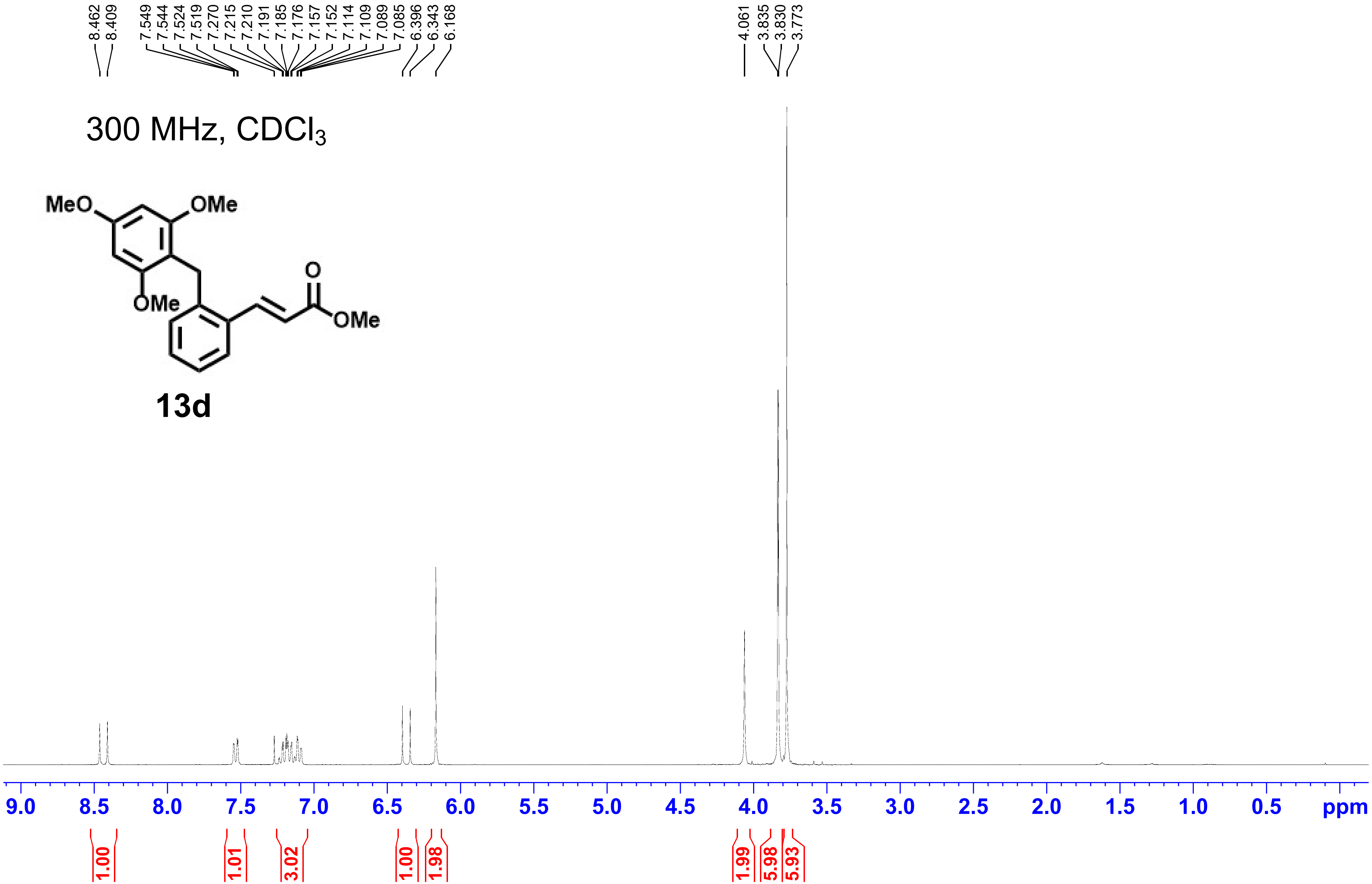

pmp.149 20, 13C, aryl fused substrate + 135-MeO3Ph, B82-300/75, 1/18/22

75 MHz, $CDCl_3$

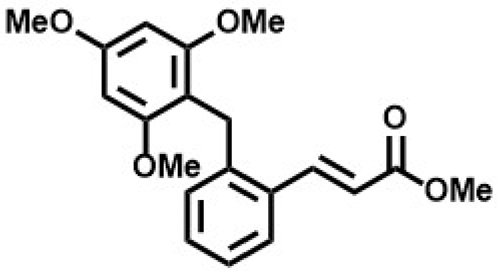

13d

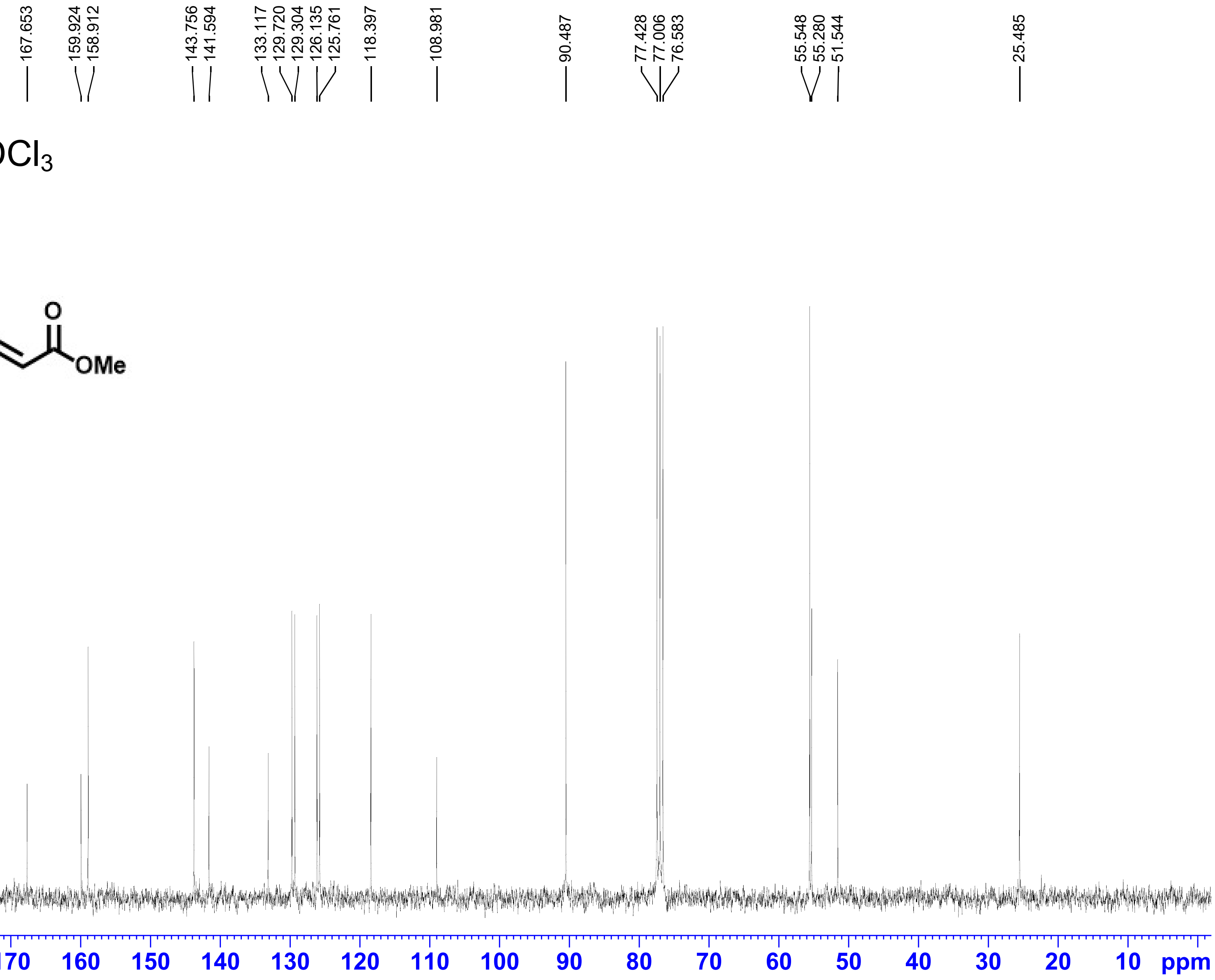

pmp.0147 20, benzo fused+ thiophene, DPX300, 2/2/22

300 MHz, $CDCl_3$

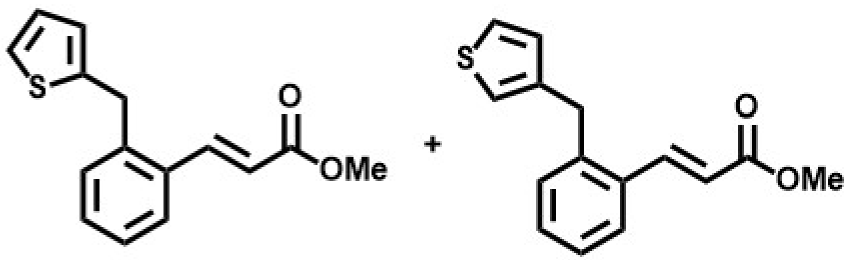

13e   13e'

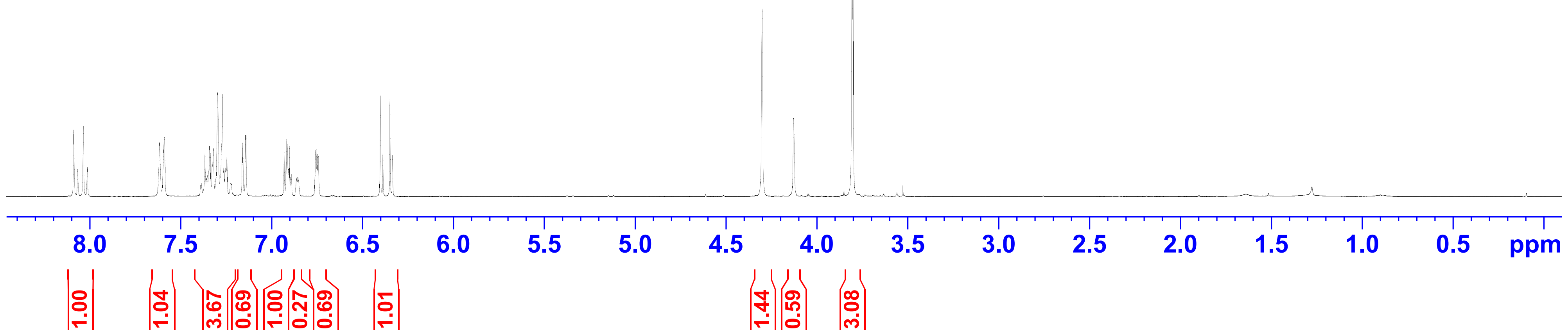

pmp.147 21, 13C, benzo fused + thiophene, DPX 300/75, 2/2/22

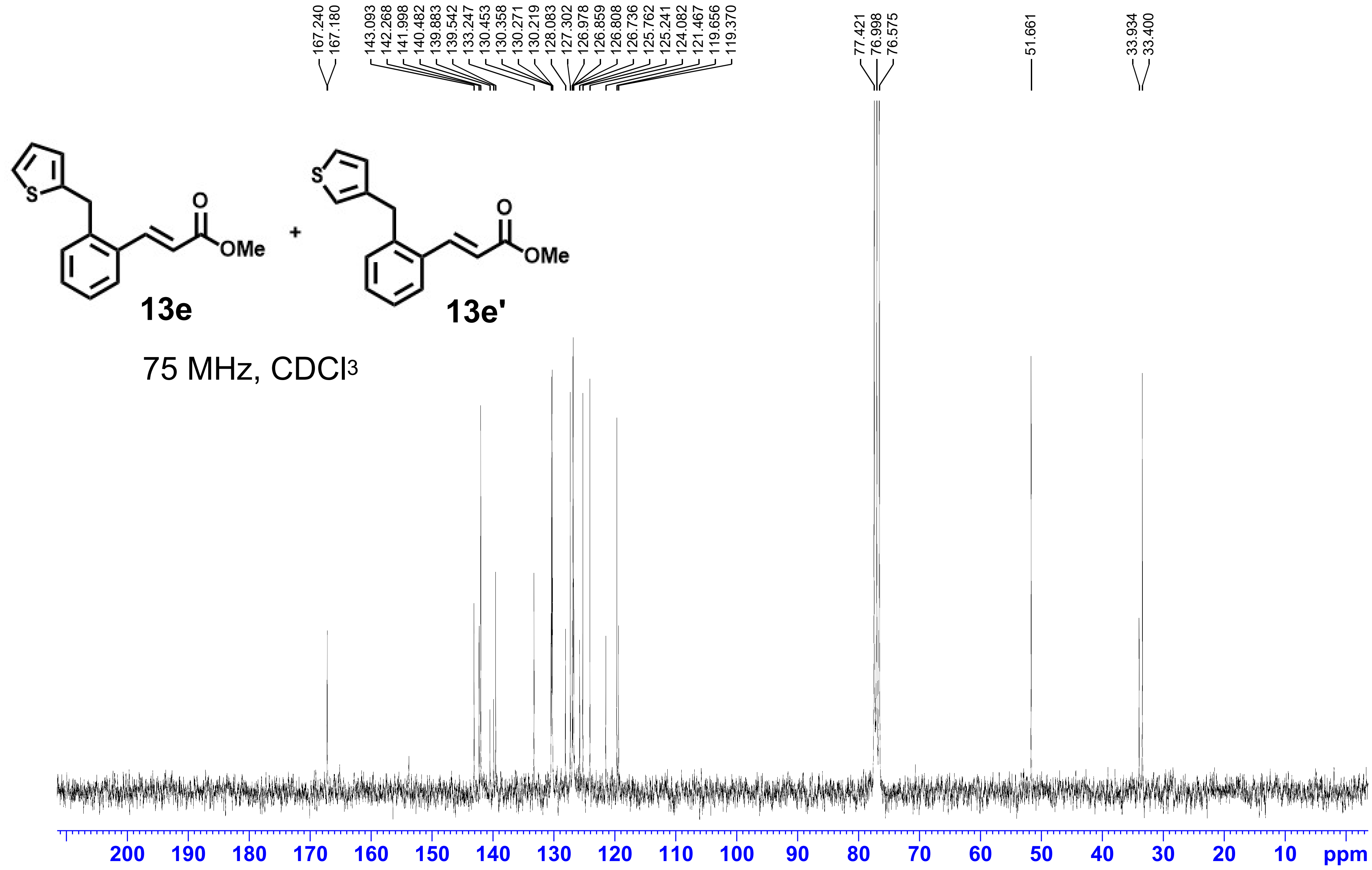

jrg.1487 2, aryl spaced cpd + GaCl3 + benzene

300 MHz, $CDCl_3$

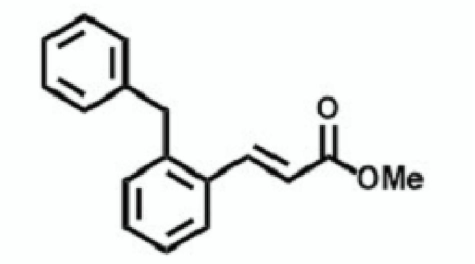

13f

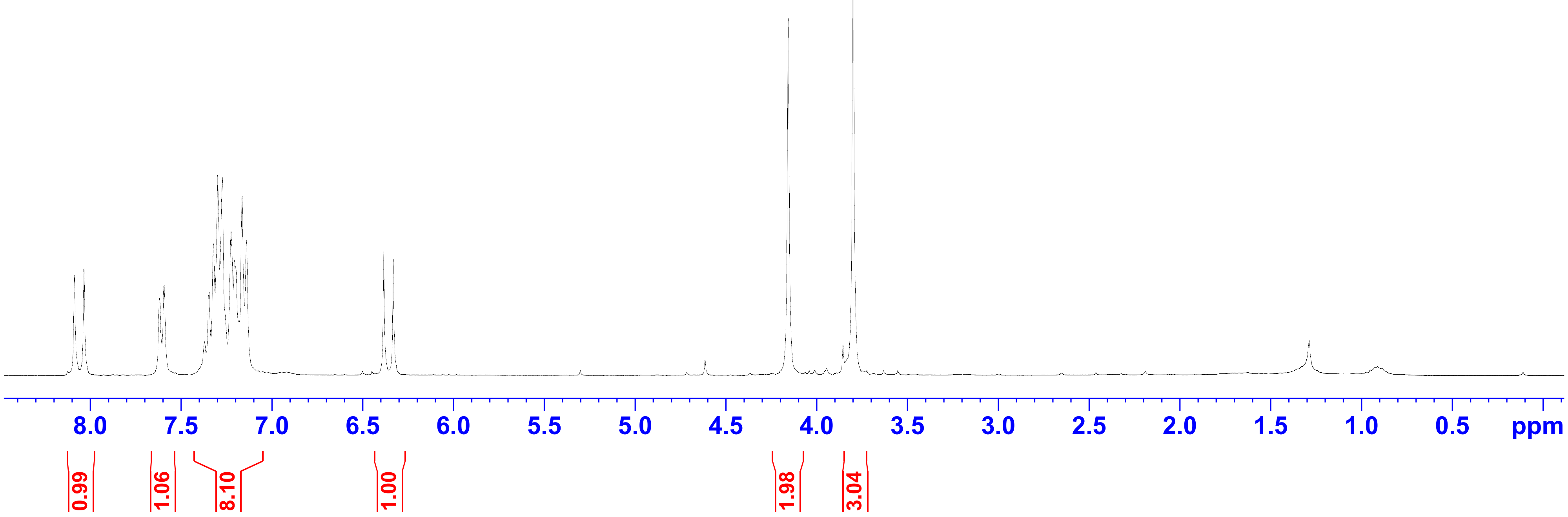

jrg.1487 21, 13C, aryl spaced substrate, Ph subst prod, 300 US/75

75 MHz, $CDCl_3$

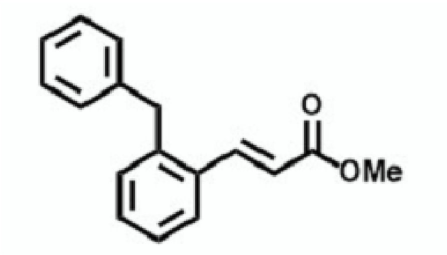

13f

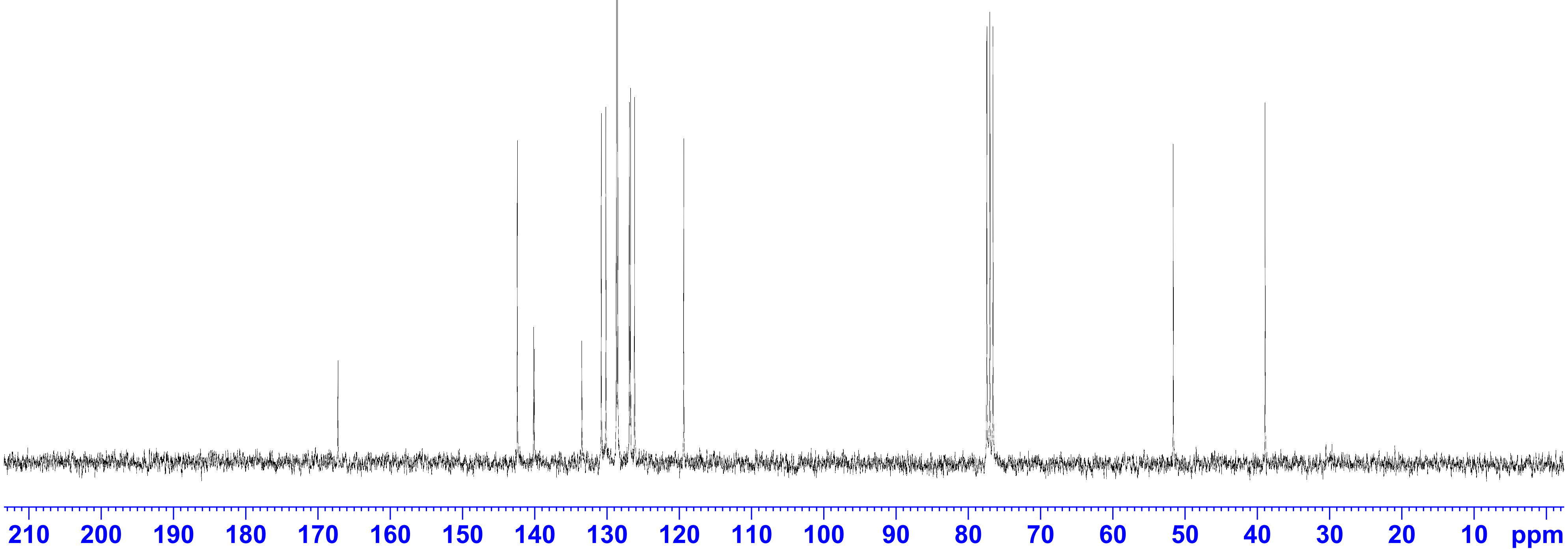

pmp.153 20, 1H, benzo + allylTMS, B82-300, 2/1/22

300 MHz, $CDCl_3$

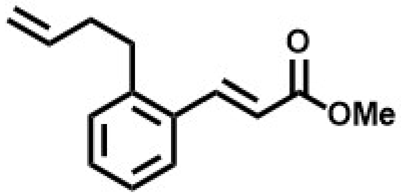

13g

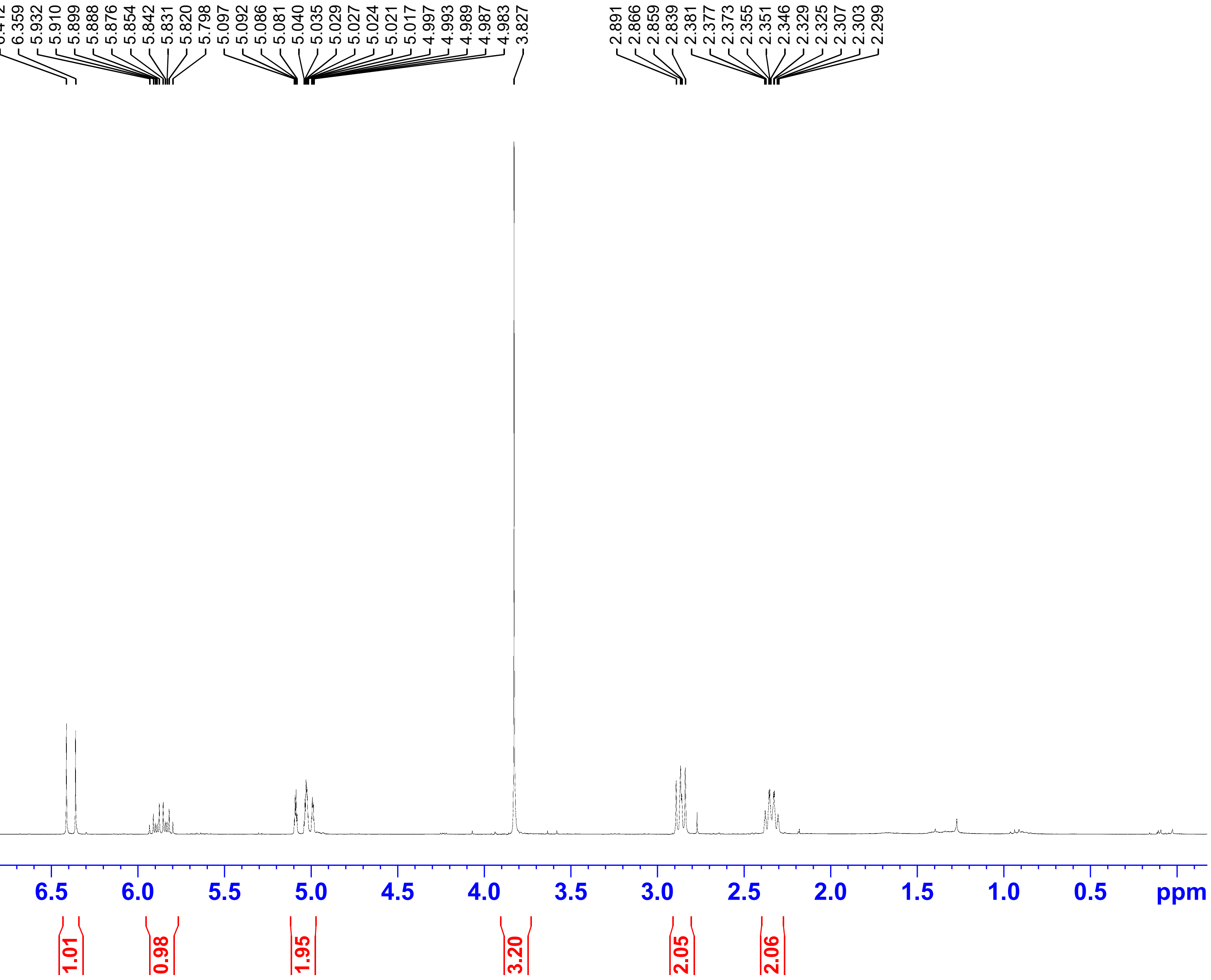

pmp.153 21, 13C, benzo fused + allylTMS, B-82 300/75, 2/1/22

75 MHz, $CDCl_3$

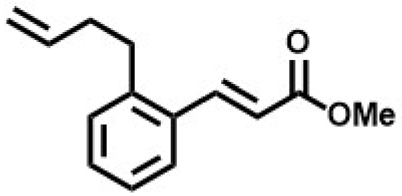

13g

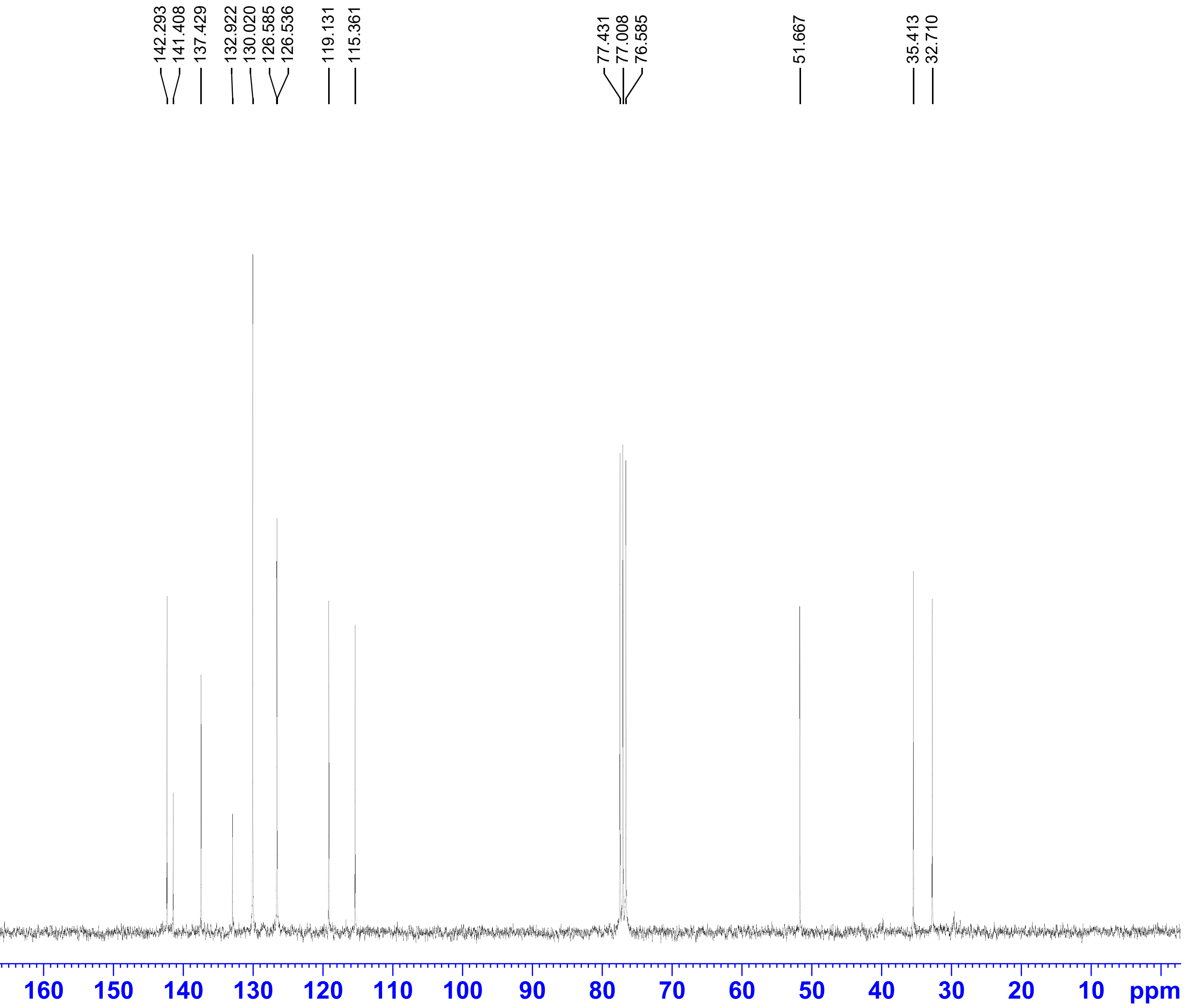

jrg.1491 20, pages Ph spaced substarte + InCl3 + propiophen Si enol, rflx, B82-300

300 MHz, $CDCl_3$

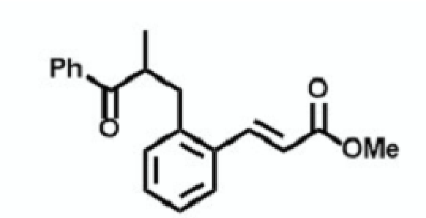

13h

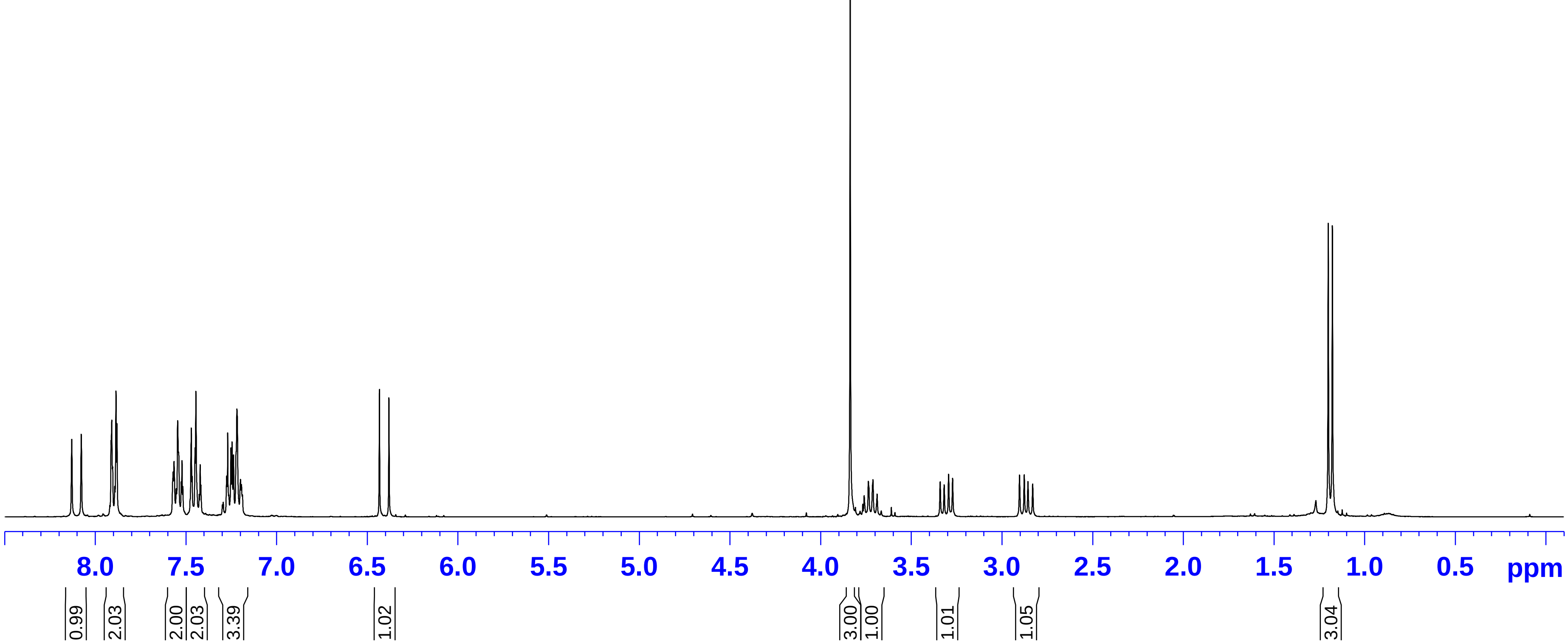

jrg.1491 21, pages Ph spaced substarte + InCl3 + propiophen Si enol, B82-300/75MHz,

300 MHz, $CDCl_3$

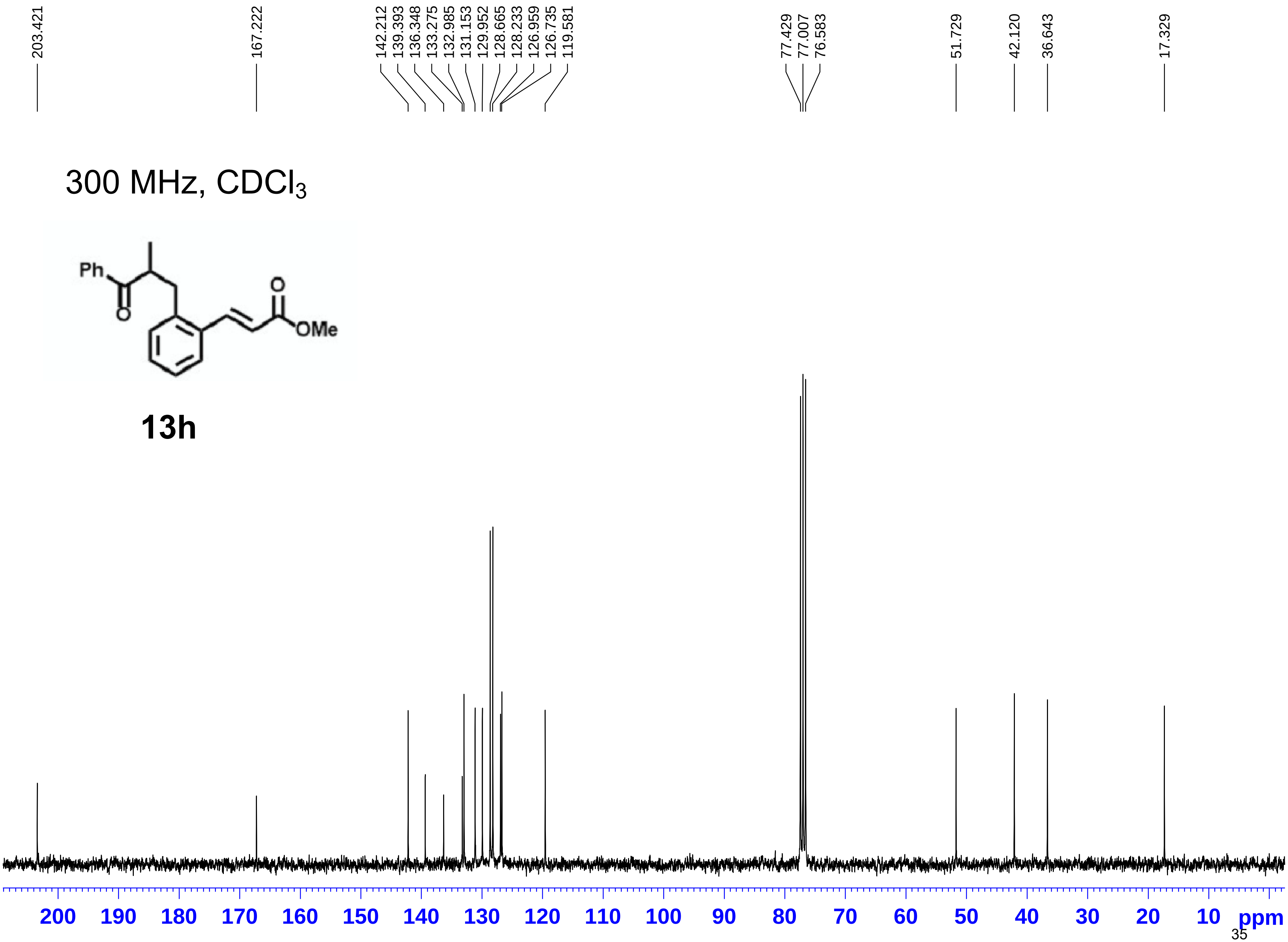

13h

Allyl bromide **7**

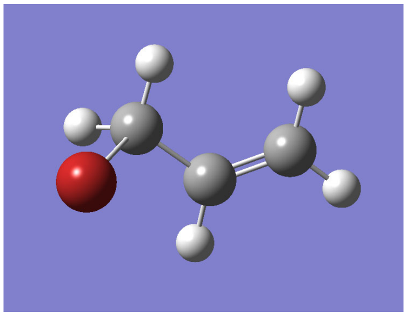

```
%chk=propene3.chk
%mem=1024MB
%nprocs=8
# opt b3lyp/6-311++g(d,p)  scrf=(solvent=dichloromethane)

propene

0 1
 Br                 -4.15384620    0.07692308    0.00000000
 C                  -6.06384620    0.07692308    0.00000000
 H                  -6.42051255    0.55052859    0.89072270
 H                  -6.42051255    0.61150859   -0.85551590
 C                  -6.57717980   -1.37411828   -0.05067148
 H                  -5.88025262   -2.18553040   -0.07900646
 C                  -7.90857136   -1.62687438   -0.05949811
 H                  -8.60997214   -0.81937431   -0.08917306
 H                  -8.25957734   -2.63740676   -0.03671404
```

Allyl Cation **7+**

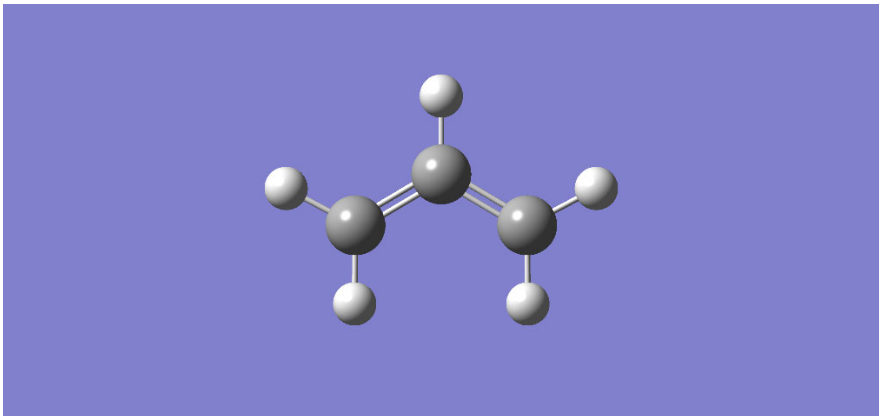

```
%chk=propeneion3.chk
%mem=1024MB
%nprocs=8
# opt b3lyp/6-311++g(d,p)  scrf=(solvent=dichloromethane)

propene

1 1
 C                 -6.06384620    0.07692308    0.00000000
 H                 -6.42051255    0.55052859    0.89072270
 H                 -6.42051255    0.61150859   -0.85551590
 C                 -6.57717980   -1.37411828   -0.05067148
 H                 -5.88025262   -2.18553040   -0.07900646
 C                 -7.90857136   -1.62687438   -0.05949811
 H                 -8.60997214   -0.81937431   -0.08917306
 H                 -8.25957734   -2.63740676   -0.03671404
```

Pentadienyl bromide **8a**

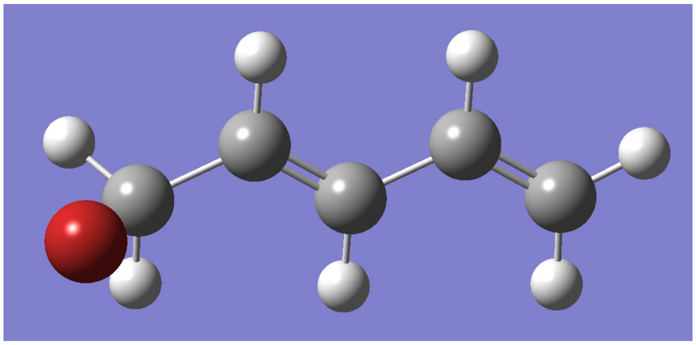

```
%chk=pentadiene3.chk
%mem=1024MB
%nprocs=8
# opt b3lyp/6-311++g(d,p)  scrf=(solvent=dichloromethane)

optimization for pentadiene

0 1
 C                  -1.84615387    0.69230768    0.00000000
 H                  -1.31299012   -0.23539724    0.00000000
 C                  -1.17087956    1.86728498    0.00000000
 H                  -1.70404331    2.79498990    0.00000000
 C                   0.36912044    1.86728498    0.00000000
 H                   0.90228419    0.93958006    0.00000000
 C                   1.05136384    3.03822946    0.00000000
 H                   0.52372137    3.96908575    0.00000000
 H                   2.12134496    3.03187216    0.00000000
 C                  -3.38615387    0.69230768    0.00000000
 H                  -3.74282021    1.19501568    0.87462780
 H                  -3.74282072    1.19840360   -0.87267157
 Br                 -4.02281996   -1.10845440   -0.00349202
```

Pentadienyl cation **8a+**

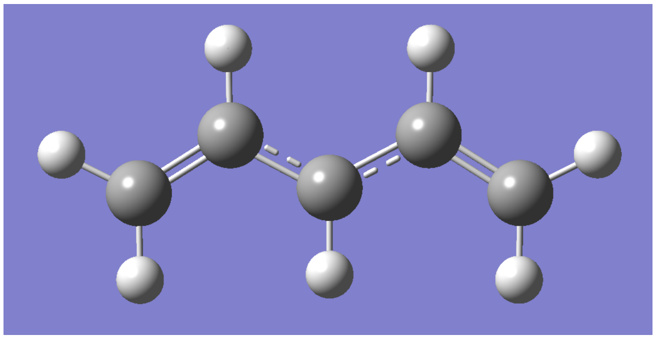

```
%chk=pentadieneion3.chk
%mem=1024MB
%nprocs=8
# opt b3lyp/6-311++g(d,p)  scrf=(solvent=dichloromethane)

optimization for pentadiene ion

1 1
 C                  -4.35233144    -0.33678756     0.00000000
 H                  -3.81916769    -1.26449248     0.00000000
 C                  -3.67705713     0.83818974     0.00000000
 H                  -4.21022088     1.76589466     0.00000000
 C                  -2.13705713     0.83818974     0.00000000
 H                  -1.60389338    -0.08951518     0.00000000
 C                  -1.45481373     2.00913422     0.00000000
 H                  -1.98245620     2.93999051     0.00000000
 H                  -0.38483262     2.00277692     0.00000000
 C                  -5.89233144    -0.33678756     0.00000000
 H                  -6.24899779     0.16930856    -0.87267166
 H                  -6.24899829     0.16592023     0.87462771
```

Pentadienyl bromide with ester **8b**

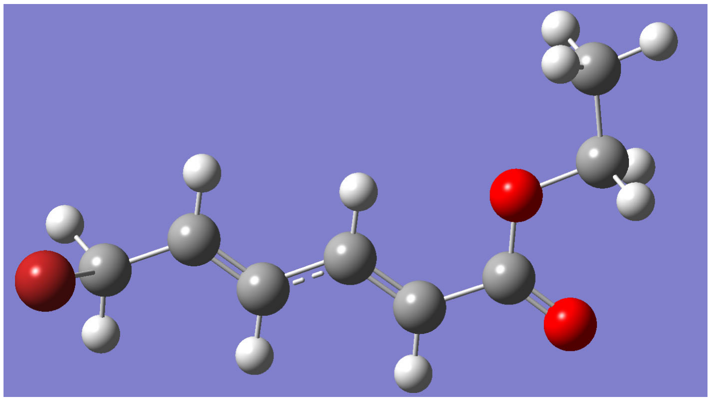

```
%chk=ethylester3.chk
%mem=1024MB
%nprocs=8
# opt b3lyp/6-311++g(d,p)  scrf=(solvent=dichloromethane)

Optimization for ethyl ester

 0 1
  C                 -0.41450776    0.25906735    0.00000000
  C                 -1.95450776    0.25906735    0.00000000
  H                 -2.48767150    1.18677227    0.00000000
  C                 -2.63675116   -0.91187713    0.00000000
  H                 -2.10910869   -1.84273341    0.00000000
  C                 -4.17672398   -0.90272737    0.00000000
  H                 -4.70436645    0.02812891    0.00000000
  C                 -4.86591238   -2.06959771    0.00000000
  H                 -4.34380982   -3.00357250    0.00000000
  O                  0.21253267    1.35011770    0.00000000
  O                  0.29803819   -0.98076259    0.00000000
  C                  1.70460386   -0.72295222   -0.00261381
  H                  1.96290100   -0.16271150   -0.87686373
  H                  1.96607541   -0.16231271    0.87043600
  C                  2.47128794   -2.05853986   -0.00370136
  H                  3.52375390   -1.86563275   -0.00519748
  H                  2.21264423   -2.61897836    0.87031930
  H                  2.21016152   -2.61898132   -0.87698159
  C                 -6.40580366   -2.05129852    0.00000000
  H                 -6.75645322   -1.54286225    0.87374908
  H                 -6.75644971   -1.54252396   -0.87355355
  Br                -7.06382257   -3.84437157   -0.00034910
```

Pentadienyl cation with ester **8a+**

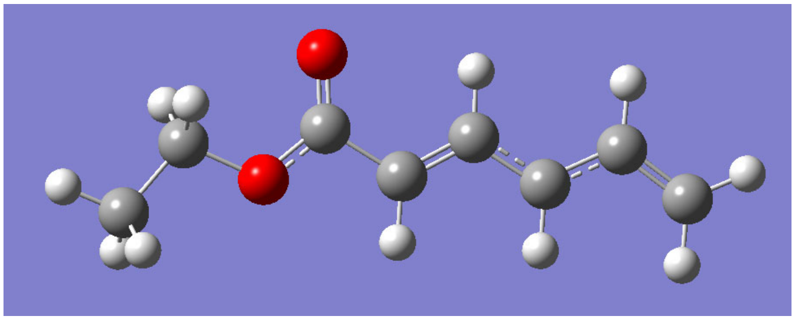

```
%chk=ethylesterion2dcm.chk
%mem=1024MB
%nprocs=8
# opt b3lyp/6-311++g(d,p)  scrf=(solvent=dichloromethane)

optimization for ethyl ester ion (no Br)

1 1
 C                 -2.07665031   -0.71377475   -1.85499308
 H                 -3.02055188   -1.17783702   -1.65853435
 H                 -1.36637849   -1.45926666   -2.14594972
 C                 -1.58063216   -0.00071868   -0.58333392
 H                 -1.40534453    1.05472597   -0.59785424
 C                 -1.36538336   -0.70540626    0.55405362
 H                 -1.53890475   -1.76104112    0.57467801
 C                 -0.86933885    0.01561205    1.82120492
 H                 -0.69581746    1.07124692    1.80058053
 C                 -0.65183749   -0.68230884    2.96233008
 H                 -0.82358650   -1.73809665    2.98905783
 C                 -0.15578412    0.04664626    4.22492879
 O                  0.04829000    1.28815179    4.20067296
 O                  0.07372218   -0.68979624    5.42903813
 C                  0.52679830    0.19927053    6.45333413
 H                 -0.21657108    0.94835583    6.62989340
 H                  1.43760273    0.66692810    6.14247776
 C                  0.77374500   -0.59447336    7.74970907
 H                  1.11276075    0.07077228    8.51614013
 H                 -0.13705969   -1.06213038    8.06056550
 H                  1.51711376   -1.34355914    7.57314922
```

Pentadienyl bromide with phenyl ketone **8c**

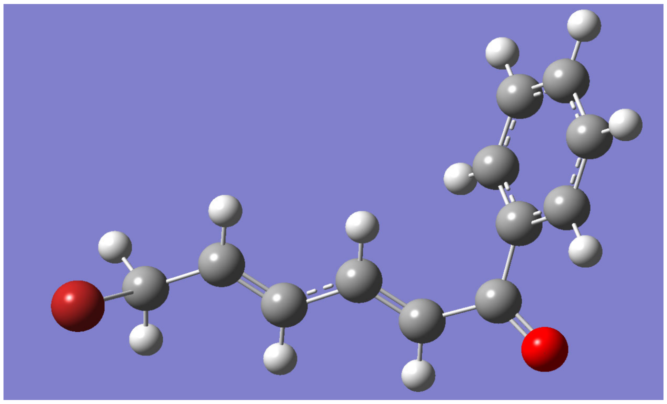

```
%chk=phenylketone3.chk
%mem=1024MB
%nprocs=8
# opt b3lyp/6-311++g(d,p)  scrf=(solvent=dichloromethane)

optimization for phenyl ketone

0 1
 Br                -5.61538468   -0.64102563    0.00000000
 C                 -7.52538468   -0.64102563    0.00000000
 H                 -7.88205103   -0.10644012   -0.85551590
 H                 -7.88205153   -1.64921670   -0.03520681
 C                 -8.03871755    0.04061221    1.28197473
 H                 -7.34178996    0.42177894    1.99884561
 C                 -9.37010899    0.15934678    1.50528168
 H                -10.07184814   -0.21986927    0.79207950
 C                 -9.87480633    0.84240444    2.78992670
 H                 -9.17306717    1.22162049    3.50312888
 C                -11.20467161    0.96485059    3.02021412
 H                -11.91119756    0.58759861    2.31070581
 C                -11.70071560    1.64930395    4.30748408
 O                -12.93412979    1.76644640    4.52779671
 C                -10.68384649    2.19226475    5.32864557
 C                 -9.31585579    2.06363210    5.08672303
 C                -11.13041427    2.81272736    6.49532954
 C                 -8.39471245    2.55477838    6.01165114
 H                 -8.96411222    1.57373472    4.16720206
 C                -10.20911971    3.30495673    7.42017356
 H                -12.20856866    2.91435312    6.68607633
 C                 -8.84144221    3.17596780    7.17860134
 H                 -7.31643193    2.45286562    5.82133187
 H                -10.56151092    3.79448432    8.33976989
 H                 -8.11496624    3.56319355    7.90770025
```

Pentadienyl cation with phenyl ketone **8c+**

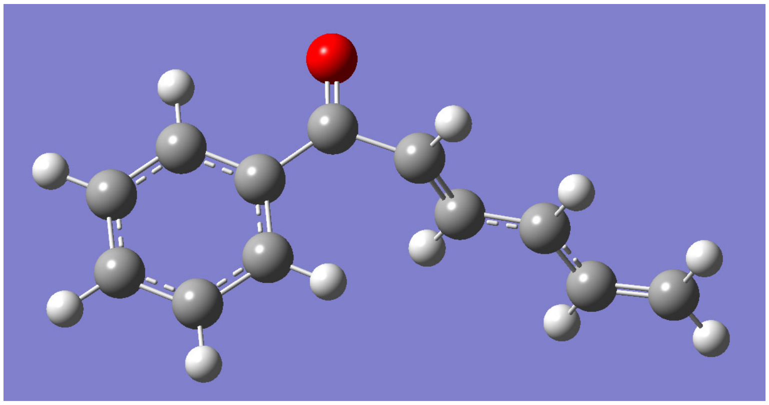

```
%chk=phenylketoneion3.chk
%mem=1024MB
%nprocs=8
# opt b3lyp/6-311++g(d,p)  scrf=(solvent=dichloromethane)

optimization for phenyl ketone ion

1 1
 C                  -7.52538468   -0.64102563    0.00000000
 H                  -7.88205103   -0.10644012   -0.85551590
 H                  -7.88205153   -1.64921670   -0.03520681
 C                  -8.03871755    0.04061221    1.28197473
 H                  -7.34178996    0.42177894    1.99884561
 C                  -9.37010899    0.15934678    1.50528168
 H                 -10.07184814   -0.21986927    0.79207950
 C                  -9.87480633    0.84240444    2.78992670
 H                  -9.17306717    1.22162049    3.50312888
 C                 -11.20467161    0.96485059    3.02021412
 H                 -11.91119756    0.58759861    2.31070581
 C                 -11.70071560    1.64930395    4.30748408
 O                 -12.93412979    1.76644640    4.52779671
 C                 -10.68384649    2.19226475    5.32864557
 C                  -9.31585579    2.06363210    5.08672303
 C                 -11.13041427    2.81272736    6.49532954
 C                  -8.39471245    2.55477838    6.01165114
 H                  -8.96411222    1.57373472    4.16720206
 C                 -10.20911971    3.30495673    7.42017356
 H                 -12.20856866    2.91435312    6.68607633
 C                  -8.84144221    3.17596780    7.17860134
 H                  -7.31643193    2.45286562    5.82133187
 H                 -10.56151092    3.79448432    8.33976989
 H                  -8.11496624    3.56319355    7.90770025
```

Et 4-bromocrotonate **9**

```
%chk=butene-ethylester.chk
%mem=1024MB
%nprocs=8
# opt b3lyp/6-311++g(d,p)  scrf=(solvent=dichloromethane)

optimization for butene with ethyl ester

0 1
 Br                 -4.15384620    0.07692308    0.00000000
 C                  -6.06384620    0.07692308    0.00000000
 H                  -6.42051255    0.55052859    0.89072270
 H                  -6.42051255    0.61150859   -0.85551590
 C                  -6.57717980   -1.37411828   -0.05067148
 H                  -5.88025262   -2.18553040   -0.07900646
 C                  -7.90857136   -1.62687438   -0.05949811
 H                  -8.60997214   -0.81937431   -0.08917306
 C                  -8.41375754   -3.08128547   -0.02670608
 O                  -9.81702773   -3.35633174   -0.03577927
 O                  -7.58885778   -4.03096591    0.00819387
 C                 -10.02631220   -4.77085091   -0.02043243
 H                  -9.59208165   -5.18606084    0.86497400
 H                  -9.56710181   -5.20871108   -0.88200327
 C                 -11.53758231   -5.06639550   -0.03821057
 H                 -11.69419467   -6.12479801   -0.02569323
 H                 -11.99695675   -4.62766303    0.82282889
 H                 -11.97163339   -4.65207125   -0.92411975
```

Gamma carbonyl cation with ester **9+**

```
%chk=butene-ethylesterion.chk
%mem=1024MB
%nprocs=8
# opt b3lyp/6-311++g(d,p)  scrf=(solvent=dichloromethane)

optimization for butene with ethyl ester

1 1
 C                 -6.06384620    0.07692308    0.00000000
 H                 -6.42051255    0.55052859    0.89072270
 H                 -6.42051255    0.61150859   -0.85551590
 C                 -6.57717980   -1.37411828   -0.05067148
 H                 -5.88025262   -2.18553040   -0.07900646
 C                 -7.90857136   -1.62687438   -0.05949811
 H                 -8.60997214   -0.81937431   -0.08917306
 C                 -8.41375754   -3.08128547   -0.02670608
 O                 -9.81702773   -3.35633174   -0.03577927
 O                 -7.58885778   -4.03096591    0.00819387
 C                -10.02631220   -4.77085091   -0.02043243
 H                 -9.59208165   -5.18606084    0.86497400
 H                 -9.56710181   -5.20871108   -0.88200327
 C                -11.53758231   -5.06639550   -0.03821057
 H                -11.69419467   -6.12479801   -0.02569323
 H                -11.99695675   -4.62766303    0.82282889
 H                -11.97163339   -4.65207125   -0.92411975
```
